# Myocardial phosphoproteomics unveils a key role of DYRK1A in aortic valve replacement-induced reverse remodelling

**DOI:** 10.1007/s00395-025-01125-w

**Published:** 2025-07-06

**Authors:** Fábio Trindade, João Almeida-Coelho, Cláudia Sousa-Mendes, Francisca Saraiva, Maria L. Arbonés, Adelino Leite-Moreira, Rui Vitorino, Inês Falcão-Pires

**Affiliations:** 1 RISE-Health, Department of Surgery and Physiology, Faculty of Medicine, University of Porto, 4200-319 Porto, Portugal; 2 IBMB-CSIC – Institut de Biologia Molecular de Barcelona, 08028 Barcelona, Spain; 3 CIBERER – Centro de Investigación Biomédica en Red de Enfermedades Raras, 08028 Barcelona, Spain; 4 iBiMED - Institute of Biomedicine , Department of Medical Sciences, University of Aveiro, 3810-193 Aveiro, Portugal; 5 LAQV/REQUIMTE, Department of Chemistry, University of Aveiro, 3810-193 Aveiro, Portugal; 6 Department of Cardiothoracic Surgery, ULS São João, 4200-319 Porto, Portugal

**Keywords:** Aortic valve stenosis, Myocardium, Reverse remodelling, Phosphoproteomics, Proteomics, DYRK1A

## Abstract

Aortic valve stenosis (AVS) is a growing healthcare burden. Aortic valve replacement (AVR) remains the only effective treatment to eliminate pressure overload and triggers myocardial reverse remodelling (RR), with regression of hypertrophy, fibrosis and diastolic function normalisation. However, many patients show an incomplete RR, being at higher risk of death. We aimed to uncover pathways and new therapeutic targets for incomplete RR through myocardial (phospho)proteomics. AVS patients were categorised based on left ventricle mass regression (LVM): complete RR (≥ 15%) or incomplete RR (≤ 5%). 83 myocardial proteins were dysregulated through LC–MS/MS. Gene ontology enrichment analysis identified inflammation, complement and immune system activation as priming events of an incomplete RR and a better mitochondrial function underscoring complete RR. Kinetic metabolic modelling corroborated the lower ATP production capacity of incomplete RR patients. To uncover therapeutic targets, kinases were predicted from phosphoproteome data. Casein kinase 2 and DYRK1A were among the most dysregulated kinases in RR. DYRK1A was found to be inversely correlated with LVM regression (*r* = − 0.62). DYRK1A functional role (passive, maximal tension and Ca^2+^ sensitivity) was evaluated in skinned cardiomyocytes from *Dyrk1a*^+/-^ mice and from AVS patients upon incubation with this kinase. Cardiomyocytes from mutant mice showed increased myofilamentary stiffness in response to stretch. Also, the raised myofilamentary stiffness of cardiomyocytes isolated from incomplete RR was normalised upon incubation with DYRK1A. Better myocardial bioenergetics may underscore a complete LVM regression. In turn, complement and immune-inflammatory pathways activation prime an incomplete response to AVR. DYRK1A emerges as a surrogate target to treat myocardial stiffness-driven diastolic dysfunction.

**Supplementary Information:**

The online version contains supplementary material available at 10.1007/s00395-025-01125-w.

## Introduction

The ever-increasing annual number of aortic valve replacement (AVR) procedures demonstrates how aortic valve stenosis (AVS) is a growing healthcare burden [1]. AVR eliminates the left ventricle (LV)’s pressure afterload imposed by the stenotic valve and triggers the process of myocardial reverse remodelling (RR), i.e. the normalisation of myocardial size, structure and function [2, 3]. Despite the haemodynamic relief, many patients fail to attain a complete RR. Instead, several patients present with postoperative LV hypertrophy. For instance, Biederman et al. [4] showed that > 85% of the subjects presented LV hypertrophy 4 years after AVR. This is a daunting scenario, considering the higher risk of adverse events, heart failure (HF) and death [5].

Age, sex, myocardial fibrosis and comorbidities, such as hypertension, obesity, coronary artery disease (CAD) or diabetes mellitus, have all been proposed as risk factors for an incomplete RR. However, different studies do not consistently reproduce the same associations and can be contradictory [6–10]. The complexity underpinning myocardial RR calls for a more granular assessment of the myocardial phenotype at the molecular level, which can enlighten the mechanisms priming an incomplete RR, and uncover new prognostic markers and therapeutic targets to maximise myocardial recovery. For now, patients showing incomplete RR can only benefit from palliative care, mainly intending to mitigate the consequences of HF.

High-throughput approaches such as proteomics have improved our understanding of the biological mechanisms underlying myocardial (reverse) remodelling. For instance, in the aortic constriction-induced pressure overload model, proteomics has evidenced cardiomyocyte hypertrophy, foetal gene programme reactivation, mitochondrial dysfunction and metabolic derangement (lipolytic to glycolytic metabolic shift) during myocardial remodelling [11, 12]. Phosphoproteomics, in particular, represents a powerful tool to identify putative therapeutic targets. For example, Chang et al. [13] showed reduced phosphorylation of Thr282 and Ser283 α-tropomyosin in aortic-banded mice, associated with reduced sarcomeric tension and ATPase activity. They also found that inhibiting Ser622 phosphorylation on dynamin-related protein 1, involved in mitochondrial fission, halted hypertrophy progression after banding.

Regarding post-AVR-induced RR, the input of proteomics is limited since most animal model studies resort to systemic hypertension as the pressure overload trigger (e.g. [14, 15]). Despite recapitulating the haemodynamic overload, hypertension is associated with remarkable neurohumoral activation, which confounds specific AVS-induced LV remodelling. Besides, those using aortic-banded mice have reported the effects of antihypertensive [16] and antioxidant therapies [17] on pressure-overloaded heart proteome and not on the aortic debanding, the best AVR model available. Notwithstanding, these studies show that it is possible to attenuate the expression of sarcomeric proteins, rescue fatty acid oxidation and restore antioxidant defences [14, 16, 17].

In the human setting, proteomics has been limited to the study of RR in patients with LV-assist devices (LVADs). As a bridge to heart transplantation, LVADs provide access to the myocardium after mechanical unloading. For instance, Weger et al. [18] demonstrated specific cytoskeleton and mitochondria sub-proteome changes upon LVAD implantation in HF patients with ischaemic heart disease or dilated cardiomyopathy. A decrease in extracellular matrix (ECM) proteins (e.g. periostin, fibulin and versican), complement system components and cardiac hormones with myocardial unloading of dilated cardiomyopathy patients was also reported [19]. Nevertheless, LVADs are used to treat patients with depressed systolic function, which is uncommon in AVS (< 20% of the patients show LV ejection fraction (LVEF) < 50%) [1]. Therefore, the insights of those proteomics studies cannot accurately be translated to the AVS setting.

Provided with the inherent difficulty in studying the molecular features of RR in AVS due to the impossibility of analysing myocardial biopsies after AVR, we can still clarify the relationship between the perioperative myocardial proteome and the patients’ outcome (e.g. LV mass regression), based on echocardiography. However, to our knowledge, no studies have been reported on myocardial proteomics in AVS patients with complete or incomplete RR. Therefore, we aimed to characterise, for the first time, the myocardial proteome and phosphoproteome in AVS patients undergoing AVR and assess its association with patients’ outcomes regarding LV mass (LVM) regression. With this holistic approach, we aimed to clarify the molecular mechanisms driving an incomplete response and to uncover new therapeutic targets to treat patients with incomplete RR.

## Methods

### Patients’ selection and clinical characterisation

AVS patients were selected based on retrospective clinical data and the myocardial samples obtained from these patients during AVR. Informed consent was obtained from all patients. This study was approved by the Ethics Committee of Centro Hospitalar Universitário de São João (Ref 109/2022) and abided by the 1964 Declaration of Helsinki and its later amendments.

Only patients undergoing AVR with severe AVS (according to ESC guidelines [20]) and with no more than one stenotic coronary vessel (stenosis > 50%) were considered. Patients with dilated or hypertrophic cardiomyopathies, previous myocardial infarction or severe cases of aortic insufficiency, mitral stenosis, mitral insufficiency or tricuspid insufficiency were excluded. Clinical evaluation of AVS severity and myocardial structure and function was performed using transthoracic Doppler echocardiography. Peak aortic valve velocity (Peak Ao), mean aortic transvalvular pressure gradient and indexed aortic valve area (AVAi) were derived from echocardiography. The mean pressure gradient was obtained with the modified Bernoulli equation and AVAi with the standard continuity equation. In turn, LV end-diastolic dimension (LVEDD), LV posterior wall thickness (PWT) and interventricular septal thickness (IVST) were derived from 2D-echocardiograms during diastole. Relative wall thickness (RWT) was calculated as 2 × PWT/LVEDD. LV mass index (LVMi) was calculated according to the recommendations for cardiac chamber quantification using the cube formula corrected by Devereux [21].

$${\text{LVM}}\, = \,0.{8 } \times \, ({1}.0{4 } \times \, \left( {\left[ {{\text{LVEDD}}\, + \,{\text{PWT}}\, + \,{\text{IVST}}} \right]^{{3}} {-}\left[ {{\text{LVEDD}}} \right]^{{3}} } \right)\, + \,0.{6} {\text{g}}$$

$${\text{LVMi}}\, = \,{\text{LVM }}/{\text{ Body Surface Area g}}/{\text{m}}^{{2}}$$

The same valve and myocardial parameters were obtained postoperatively by follow-up echocardiography to assess the prosthesis’ haemodynamic performance, the existence of patient–prosthesis mismatch (PPM) and RR. According to the American Society of Echocardiography, a postoperative mean gradient < 20 mmHg, between 20 and 34 mmHg, and ≥ 35 mmHg was defined as normal, possible stenosis and significant stenosis [22]. A mild-to-moderate mismatch was defined as an effective orifice area < 0.85 cm^2^/m^2^ and a severe mismatch if < 0.65 cm^2^/m^2^, according to Pibarot and Dumesnil [23]. RR evaluation was based on the assessment of LV hypertrophy. LVM regression (%) was defined as the difference between pre- and postoperative LVMi. Patients with LVM regression ≥ 15% (i.e. above the median regression of a population initially screened for this study, composed of 77 patients, Fig. S1) and ≤ 5% (i.e. below the first quartile for the same population), respectively, composed the complete RR (cRR) and incomplete RR (iRR) groups. This 10% gap was included to emphasise the phenotypical differences between the subpopulations.

All patients enrolled in this study were free of dilated or hypertrophic cardiomyopathies. Upon preoperative echocardiographic assessment, all subjects showed left ventricle ejection fraction (LVEF) > 50% or had a written record of normal/good systolic function.

Eight patients were selected for an exploratory shotgun proteomics/phosphoproteomics study (discovery cohort, A). 14 subjects were used for validation immunoblots experiments (validation cohort, B), and 12 to isolate cardiomyocytes from myocardial biopsies (functional studies, C).

### Sample collection and processing

LV myocardial biopsies were collected during AVR and immediately frozen at − 80 °C. Biopsy material consisted of endomyocardial tissue resected from the LV outflow tract (Morrow procedure) because of concomitant LV outflow tract hypertrophy, not explained by hypertrophic (obstructive) cardiomyopathy. The tissue was homogenised, and the protein was extracted with a lysis buffer (10 µL/mg tissue) using 1.4 mm zirconium oxide beads in a bead-beating system (Precellys, Bertin Instruments). The lysis buffer was composed of 7.1 M urea, 45 mM HEPES pH 8, and supplemented with 1 mM EDTA, protease inhibitors (1.2 mM AEBSF, 0.46 µM aprotonin, 14 µM bestatin, 12.3 µM E-64, 112 µM leupeptin and 1.16 µM pepstatin, mammalian cocktail VWR^®^) and phosphatase inhibitors (PhosStop, Roche^®^). Tissue grinding and protein extraction were carried out in two cycles of 30 s at 6500 rpm, with a 5-min interval for cooling on ice. Samples were then centrifuged at 12,000 rpm, 4 ºC for 15 min to remove debris, and the supernatants were collected and immediately stored at − 80 °C until further processing. Protein concentration was determined by the bicinchoninic acid method (Pierce^®^, Thermo Scientific), using bovine serum albumin as standard.

### Myocardial proteome and phosphoproteome analysis

#### In-solution digestion, peptide cleanup and phosphopeptide enrichment

For each sample, 500 µg of protein was reduced with 1500 nmol of dithiothreitol (DTT, 1 h, 37 °C) and alkylated in the dark with 3000 nmol iodoacetamide (IAA, 30 min, 25 °C). The resulting protein extract was first diluted 1:3 with 200 mM NH_4_HCO_3_, digested with 50 µg LysC (Wako, cat #129–02541) overnight at 37 °C, diluted 1:2 and digested further with 50 µg of trypsin (Promega, cat #V5113) for 8 h at 37 ºC. The peptide mix was acidified with formic acid (FA) and desalted with a MacroSpin C18 column (The Nest Group, Inc). 5 µg was reserved for the proteome analysis, and 495 µg of each sample was enriched in phosphopeptides with the Pierce TiO_2_ Phosphopeptide Enrichment kit (Thermo Scientific, cat #88301).

#### LC–MS/MS analysis

The peptide and phosphopeptide mixes were analysed in different runs, using an Orbitrap Fusion Lumos mass spectrometer (Thermo Scientific, San Jose, CA, USA) coupled to an EasyLC (Thermo Scientific (Proxeon), Odense, Denmark). Peptides were loaded directly onto the analytical column and separated by reversed-phase chromatography using a 50-cm column with an inner diameter of 75 μm, packed with 2 μm C18 particles (Thermo Scientific, San Jose, CA, USA). Chromatographic gradients were started at 95% buffer A (0.1% FA in water) and 5% buffer B (0.1% FA in acetonitrile) with a flow rate of 300 nL/min and gradually increased to 22% buffer B in 79 min and then to 35% buffer B in 11 min. A 10-min wash with 5% buffer A and 95% buffer B was performed after each run.

The mass spectrometer was operated in DDA mode, and full MS scans with 1 micro scan at a resolution of 120.000 were used over a mass range of *m/z* 350–1500 with detection in the Orbitrap. Auto gain control (AGC) was set to 2e5 and dynamic exclusion to 60 s. In each cycle of DDA analysis, following each survey scan, top speed ions with charges 2–7 above a threshold ion count of 1e4 were selected for fragmentation at a normalised collision energy of 28%. Fragment ion spectra produced via high-energy collision dissociation were acquired in the Ion Trap. AGC was set to 3e4; isolation window of 1.6 m*/z* and a maximum injection time of 40 ms were used. All data were acquired with Xcalibur software v3.0.63.

#### Data analysis

The MaxQuant software suite (v.1.6.0.16), using the Andromeda search engine, was used for peptide identification and quantification. Spectra were searched against the human proteins in the SwissProt database (release April 2018). Mass tolerance for the precursor and fragment ion was set, respectively, to 4.5 ppm and 0.5 Da. Enzyme specificity was set to trypsin, not allowing more than three missed cleavages. Carbamidomethylation on cysteines was defined as a fixed modification, whereas methionine oxidation, N-terminal acetylation and phosphorylation of serine, threonine or tyrosine were specified as variable modifications. Proteins were quantified by the MaxLFQ algorithm. Identified peptides were filtered using a 1% FDR. Only proteins identified with two or more peptides were considered.

### Bioinformatics analysis

#### Analysis of dysregulated proteins and phosphopeptides

MaxQuant’s label-free quantification algorithm (MaxLFQ) was used to calculate the fold-change of proteins/phosphopeptides between cRR and iRR. MaxLFQ normalises data based on peptide intensities [24]. Contaminant proteins, reversed sequences and proteins/peptides identified only by site were removed, data were log2-transformed and the differentially expressed proteins/peptides (DEPs) were identified by t-testing. Only phosphopeptides identified in at least three patients per group were considered. As an additional validation step of the DEPs, the effect size (standardised mean difference or Cohen’s d) was calculated.

#### Gene ontology enrichment analysis

A first gene ontology enrichment analysis (GOEA) of the biological processes (BP), molecular functions (MF) and cellular components (CC) was performed with the R package clusterProfiler [25]. For this analysis, the DEPs and cRR- and iRR-exclusive proteins were selected. Only the terms with a significant enrichment (*p* < 0.05) and passing a 5% FDR filter were considered. To provide a deeper screen of the dysregulated biological processes in (in)complete RR and of the protein–protein interactions, a second GOEA was performed with Cytoscape’s plugins ClueGO (v.2.5.3) [26] and Cluepedia (v.1.5.3). For this purpose, two clusters were defined (cRR and iRR) comprising the same proteins. The GO range was fixed between levels 5 and 15, and a minimum of three genes per cluster was defined. The GO library was updated on January 22, 2019. Dysregulated GO terms were determined by a hypergeometric test, and the p-values were adjusted using the Bonferroni step-down method.

#### In silico metabolic profiling

To explore the metabolic preferences in the myocardium of cRR and iRR patients, we used a kinetic model of cardiac metabolism, Quantitative System Metabolism^™^ (Doppelganger, Germany) [27]. Proteome quantification data (intensity profiles) were used to scale the maximal activities of enzymes and transporters, assuming proportionality with their abundance. The metabolic response to additional workload (increasing energetic demand) was modelled to determine substrate preference, oxygen uptake and ATP production rates. For the calculations, we set an overnight fasting condition with the following metabolite concentrations: 5.8 mM glucose, 0.5 mM fatty acids, 0.8 mM lactate, 0.2 mM valine, 0.15 mM leucine, 0.06 mM isoleucine, 0.08 mM β-hydroxybutyrate, 0.04 mM acetoacetate, 0.75 nM catecholamines and 100 pM insulin.

#### Motif analysis and kinase prediction

Motif analysis was performed with WebLogo (https://weblogo.berkeley.edu/logo.cgi) tool, using the differentially expressed phosphopeptides and setting six flanking amino acid residues in each of the phosphosite’s *termini*. To compensate for entropy overestimation, the small sample correction feature was used. Group-based Prediction System (GPS) 3.0 tool was used to predict dysregulated kinases [28]. The species-specific (*Homo sapiens*) and the dual-specificity (designed for kinases that can phosphorylate Ser/Thr and Tyr residues) modalities were used. Only phosphosites with a probability of occurrence > 0.9 (defined by MaxLFQ) were considered, and the prediction was set to the highest threshold available (a false positive rate of 2% and 4%, respectively, for Ser/Thr and Tyr kinases). Predicted kinases were ranked according to the difference in the percentage of all phosphorylation events associated with cRR and iRR. The kinases at the first and tenth percentile (corresponding to a difference in assigned phosphorylation events ≥ 0.9%) were deemed the most associated/active in cRR and iRR. Finally, network analysis was undertaken with Cytoscape v.3.7.1 to decipher kinase–substrate interactions. The top three kinases in each group and the respective phosphosites were selected for this analysis. The phosphosite’s node size was defined to represent the fold-change of variation between complete and incomplete RR.

### Western blot validation

Some DEPs and predicted kinases were selected for western blot validation in a larger AVS population. The specific blot conditions, solutions and antibody details are compiled in Table S1. 30 µg of protein obtained from myocardium lysates was resolved by SDS-PAGE (12%, except for complement C3: 10%), essentially as described by Laemmli [29]. Due to the high urea concentration, even after mixing with the loading buffer (3.6 M final), myocardial samples were incubated at 37 °C for 10 min to prevent non-enzymatic protein carbamylation and an undesired band mobility shift. Gels were run at constant voltage: 120 V for 10 min and 200 V for about an hour. Next, proteins were blotted onto 0.45 µm nitrocellulose membranes (Amersham^™^ Protan^™^, GE Healthcare) in transfer buffer (25 mM Tris(hydroxymethyl)aminomethane (Tris), 192 mM glycine, pH 8.3, 20% methanol V/V) for 2 h at 200 mA. Protein loading was normalised by Ponceau S staining (0.1% in 5% acetic acid V/V) because previous studies demonstrated that total protein detection is more reliable than commonly used housekeeping proteins (glyceraldehyde 3-phosphate dehydrogenase, α- and γ-tubulins, α-actinin and β-actin) to control protein loading in hypertrophy models [30]. The membranes were blocked with non-fat milk. Then, they were incubated with the specific primary antibody, washed thrice with TBS-T for 10 min, incubated with the respective secondary horseradish peroxidase-linked antibody and rewashed thrice with TBS-T for 10 min. The detection was carried out with enhanced chemiluminescence (Western Bright^™^, Advansta) using the ChemiDoc^™^ Touch Imaging System. Blot scans were analysed with ImageLab 5.1 software (Bio-Rad). Each band’s optical density (OD) was normalised to the lane’s total OD, obtained with Ponceau S staining and then to the technical control.

### Immunofluorescence and histology

Immunofluorescence was used to inspect the intracellular distribution of DYRK1A in myocardial tissue (Fig. S2, top panel). The extent of myocardial fibrosis was also assessed by Picrosirius red staining (Fig. S2, bottom panel). Essentially, myocardial biopsies (*n* = 9) were fixed with 10% (V/V) buffered formalin, processed and included in a paraffin block. Serial Sects. (3 µm thick) were cut using a microtome and mounted on silane-coated slides. For immunofluorescence, slides were rinsed three times with phosphate-buffered saline (PBS, Sigma), blocked with 1% bovine serum albumin (Fisher Scientific) for 10 min and rinsed again three times with PBS. Then, sections were incubated for 1 h with anti-DYRK1A antibody fluorochrome FITC (1:100 dilution, Biorbyt, orb461596) at room temperature and washed again thrice with PBS. The nuclei were stained by incubating the slides with 4′,6-diamidino-2-phenylindole (DAPI Fluoromount-G, Biorbyt, #0100–20) for 5 min at room temperature. Images were taken with a fluorescence microscope (Zeiss Axio Imager.Z1 Apotome), and the intensity was extracted with ImageJ-Fiji software (version 2.9.0). To assess fibrosis, the slides stained with Picrosirius red were visualised with an optical microscope (Zeiss AXIO Scope.A1) and the images were analysed with the Image Pro Plus software (version 6.0).

### Dissecting DYRK1A’s functional role

To explore the functional role of DYRK1A in the myocardium, the myofibrillar stiffness and myofilamentary calcium sensitivity were assessed in permeabilised cardiomyocytes isolated from *Dyrk1a*^+/-^ and wild-type littermate mice [31]. To translate the findings to the human setting, cardiomyocytes obtained from 12 human myocardial biopsies (6 cRR and 6 iRR) were additionally treated with recombinant DYRK1A to assess its effect on myofibrillar stiffness.

#### Animals

*Dyrk1a*^+/-^ and wild-type littermate mice (3 males and 2 females in each genotype) were included in the study. *Dyrk1a*^+/-^ mice were maintained in a C57BL/6;129S2 genetic background and genotyped as described elsewhere [31]. The animals were housed in the CID-CSIC animal facility with food and water supplied ad libitum at approximately 20 °C, 60% humidity and a 12-h light/dark cycle. Six-week-old mice were killed by experienced personnel using carbon dioxide (CO_2_) inhalation. Mice were placed in a euthanasia chamber that was then filled with 100% CO_2_ to displace the air from the chamber at a filling rate of approximately 50% of chamber volume per minute. The heart was harvested and frozen in dry ice.

All work with animals was carried out in accordance with the guidelines from Directive 2010/63/EU of the European Parliament on the protection of animals used for scientific purposes using protocols approved by the CSIC Ethics Committee and by the local government, The Generalitat de Catalunya (Experimental Project num. 9486).

#### Isolation and permeabilisation of cardiomyocytes

The detailed protocol for skinned cardiomyocyte preparation has been described elsewhere [32]. Briefly, around 5 mg of mice or human myocardial samples were defrosted in cold relaxing solution (5.95 mM Na_2_ATP, 6.04 mM MgCl_2_.6H_2_O, 2 mM (titriplex) ethylene glycol-bis(2-aminoethyl ether)-N,N,N',N'-tetraacetic acid, EGTA, 139.6 mM KCl and 10 mM imidazole) and mechanically disrupted in a Potter–Elvehjem glass, using a tissue grinder paced at 30–40 rpm (three strokes, 2 s each). Triton® X-100 10% was added to the cell suspension to a final concentration of 0.1%, and the cells were incubated for 5 min to remove the membrane structures (Fig. S3). To completely remove the detergent, the skinned cardiomyocytes were washed five times with a cold relaxing solution and pelleted by centrifugation (1 min, 4 °C, 1,500 rpm).

#### Force measurements and calcium sensitivity

Skinned cardiomyocytes suspended in a relaxing solution were poured onto coverslips and the cells were allowed to set. Single rod-shaped cardiomyocytes showing a good striation pattern were attached with silicon glue (Marineland, #31003) between a force transducer and an electromagnetic motor on an inverted microscope. Two subsequent protocols were followed. In the first, sarcomere length–passive tension (SL–-PT) relationships between 1.8 and 2.3 µm were acquired at 15 °C, with 0.1 µm step increases to assess myocardial stiffness. In the latter, the sarcomere length was set at 2.2 µm, and the development of active force was monitored indirectly by assessing total force (F_active_ = F_total_–F_passive_) in activating solutions with different calcium concentrations (pCa: 5.0, 5.2, 5.4, 5.6, 5.8 and 6.0; diluted from a stock of 5.97 mM Na_2_ATP, 6.28 Mm MgCl_2_, 40.64 mM propionic acid, 100 mM N,N-Bis(2-hydroxyethyl)taurine, 7 mM Ca-EGTA and 14.5 mM phosphocreatine disodium salt hydrate, Na_2_PCr). Passive force was measured through a “slack” test (cells were shortened to 80% of the initial length) in relaxing solution (5.89 mM Na_2_ATP, 6.48 mM MgCl_2_, 6.97 mM EGTA, 40.76 mM propionic acid, 100 mM N,N-Bis(2-hydroxyethyl)taurine and 14.5 mM phosphocreatine disodium salt hydrate, Na_2_PCr). Several cardiomyocytes from each animal or human were used, and the data were pooled in each case (*Dyrk1a*^+*/*+^ vs *Dyrk1a*^+/-^; cRR vs iRR).

#### DYRK1A effect on myofibrillar stiffness

To assess the effect on the passive force, a surrogate of myofibrillar stiffness, human-derived cardiomyocytes were additionally treated with recombinant DYRK1A (PV3785, lot:1783084L, Thermo Fisher). Cardiomyocyte DYRK1A intake was confirmed in control experiments by immunofluorescence (Fig. S4). The SL–PT relationships were obtained for each cell before and after incubation with DYRK1A (3.3 ng/µL, maximal activity according to the manufacturer) and diluted in relaxing solution, containing 6 mM dithiothreitol. A 20-min incubation was defined after testing the effect of different incubation times (10, 20, 30, 40 min) on passive tension reduction.

#### Data analysis

Force recordings were analysed with an in-house created program (Cellprog) to extract data on total, passive and active tensions, rate of force redevelopment (k_tr_) and residual force. Force was normalised for myocyte cross-sectional area, which was calculated after measuring width and length, assuming an elliptical shape of the cell. A modified Hill equation (below) was used to fit the (sigmoidal) relationship between relative active force (fraction of the maximal force obtained at pCa 4.5, F_relative_) and pCa. With this equation, the cooperativity (nHill) and the myofilamentary calcium sensitivity (pCa_50_, the midpoint of the curve, corresponding to the pCa when the developed force is half of the maximal) were obtained.

$${F}_{relative}\left({Ca}^{2+}\right)=\frac{{{[Ca}^{2+}]}^{nHill}}{{{[pCa}_{50}^{2+}]}^{nHill}+{{[Ca}^{2+}]}^{nHill}}.$$

### Statistical analysis

Categorical clinical data are presented as absolute frequencies. Fisher’s exact test was applied to detect differences between groups (cRR and iRR). Continuous demographical data, molecular expression data and force measurements are presented as mean ± SD. The normality of the distribution was tested using the D’Agostino and Pearson omnibus method. Total, passive and active tensions were log-transformed to obtain a normal distribution. The molecular differences between groups in normally distributed variables were tested with the unpaired two-tailed t-test, otherwise, the Mann–Whitney test was used. The correlation between clinical parameters and the relative protein expression was evaluated using Pearson’s or Spearman’s test, depending on whether the data presented a normal or non-normal distribution. Statistical tests were conducted with GraphPad Prism 6, and *p* < 0.05 was considered significant.

## Results

All included subjects presented severe AVS. In all cases, the aortic valve area was ≤ 0.60 cm^2^/m^2^ or the mean transvalvular pressure gradient was ≥ 40 mmHg (Table 1). Overall, the penetrance of common risk factors, such as hypertension, diabetes mellitus, CAD, smoking history or dyslipidaemia, was similar between cRR and iRR patients. Although iRR patients had a significantly higher BMI than cRR in the discovery cohort (A), this difference was absent in the validation groups (B and C). Patients with iRR were mostly females in cohorts A and B, but with no statistical difference. No differences were observed regarding preoperative and postoperative pharmacotherapy, periprosthetic gradients (mean and max) and effective orifice area. No patients presented severe PPM, and only two presented mild-to-moderate PPM. Concerning the myocardium, the study population presented with a typical concentric remodelling pattern, with preservation of systolic function in all patients. Therefore, the (phospho)proteome differences herein presented essentially reflect a higher (cRR) or lower (iRR) proclivity to post-AVR LVM regression.

**Table 1:** Clinical data of the study subpopulations

Subpopulations									
Subpopulations	A—Discovery phospho/proteomics			B—Western blot validation			C—Functional studies		
Parameters									
Degree of reverse remodelling	Complete (∆LVM ≥ 15%)	Incomplete (∆LVM ≤ 5%)	*p*	Complete (∆LVM ≥ 15%)	Incomplete (∆LVM ≤ 5%)	*p*	Complete (∆LVM ≥ 15%)	Incomplete (∆LVM ≤ 5%)	*p*
Demographics									
*N*	4	4		7	7		6	6	
Age (mean ± SD)	75.5 ± 8.6	65.5 ± 8.2		69.7 ± 10.6	72.9 ± 9.5		72.3 ± 3.8	76.3 ± 4.0	
Sex (n male: n female)	2:2	0:4		4:3	1:6		5:1	3:3	
BMI (kg/m^2^) (mean ± SD)	25.3 ± 2.3	37.0 ± 6.4	*	30.2 ± 9.7	32.7 ± 7.9		30.4 ± 4.2	25.9 ± 5.3	
Hypertension (*n*)	4	3		6	6		4	5	
Diabetes mellitus (*n*)	3	1		5	4		1	0	
CAD (≤ 1 vessel) (*n*)	0	1		1	1		1	1	
Smoking history (*n*)	1	0		2	1		2	1	
COPD (*n*)	1	1		1	1		0	0	
Dyslipidaemia (*n*)	3	3		6	7		4	5	
Mild-to-moderate aortic insufficiency (*n*)	4	3		6	5		5	4	
Mild-to-moderate mitral stenosis (*n*)	0	0		0	0		1	0	
Mild-to-moderate mitral insufficiency (*n*)	4	4		6	5		5	5	
Mild-to-moderate tricuspid insufficiency (*n*)	3	3		6	4		5	5	
**Pharmacology (preoperative)**									
Statins (*n*)	3	3		6	7		3	6	
Beta-blockers (*n*)	1	1		4	1		2	2	
ACEi (*n*)	1	1		3	2		1	1	
AT2Ri (*n*)	1	1		2	4		2	3	
Diuretics (*n*)	4	3		6	6		3	5	
MRA (*n*)	1	0		1	0		0	1	
Antiaggregants (*n*)	2	1		5	4		2	3	
Anticoagulants (*n*)	2	0		2	0		3	1	
**Preoperative echocardiographic parameters**									
Ejection fraction > 50% (*n*)	4	4		7	7		5^a^	5	
AVAi, cm^2^/m^2^ (mean ± SD)	0.43 ± 0.09	0.45 ± 0.09		0.41 ± 0.09	0.43 ± 0.08		0.45 ± 0.10	0.45 ± 0.15	
Max ATPG, mmHg (mean ± SD)	93.5 ± 28.1	79.0 ± 18.7		88.1 ± 23.0	81.6 ± 19.3		92.0 ± 12.7	86.0 ± 24.7	
Mean ATPG, mmHg (mean ± SD)	56.8 ± 16.5	49.8 ± 9.5		54.0 ± 13.3	52.6 ± 13.9		57.0 ± 9.3	51.0 ± 17.4	
LVEDD, cm (mean ± SD)	5.2 ± 0.2	5.1 ± 0.4		5.1 ± 0.4	4.6 ± 0.6		4.9 ± 0.6	4.6 ± 0.3	
IVST, cm (mean ± SD)	1.4 ± 0.1	1.3 ± 0.2		1.4 ± 0.2	1.4 ± 0.3		1.7 ± 0.3	1.1 ± 0.2	**
PWT, cm (mean ± SD)	1.3 ± 0.1	1.2 ± 0.1		1.3 ± 0.1	1.1 ± 0.2		1.5 ± 0.2	1.0 ± 0.1	***
RWT (mean ± SD)	0.48 ± 0.04	0.48 ± 0.08		0.51 ± 0.07	0.50 ± 0.14		0.60 ± 0.14	0.41 ± 0.07	*
LVMi, g/m^2^ (mean ± SD)	174.5 ± 29.9	144.8 ± 21.4		162.5 ± 36.9	130.9 ± 23.2		184.1 ± 40.6	101.8 ± 12.9	***
**Pharmacology (postoperative)**									
Statins (*n*)	3	3		5	7		3	6	
Beta-blockers (*n*)	2	4		4	7		5	5	
ACEi (*n*)	1	1		2	3		2	1	
AT2Ri (*n*)	0	0		0	1		0	2	
Diuretics (*n*)	4	3		7	7		5	6	
MRA (*n*)	0	0		0	0		0	0	
Antiaggregants (*n*)	1	2		1	4		1	5	
Anticoagulants (*n*)	3	3		6	5		5	1	
**Postoperative echocardiographic parameters**									
Ejection fraction > 50% (*n*)	3	4		6	7		6	6	
EOAi, cm^2^/m^2^ (mean ± SD)	1.23 ± 0.31	0.99 ± 0.14		1.20 ± 0.31	1.08 ± 0.31		1.20 ± 0.18	1.17 ± 0.32	
Mild–moderate patient–prosthesis mismatch (*n*)	0	0		0	1		0	1	
Severe patient–prosthesis mismatch (*n*)	0	0		0	0		0	0	
Max ATPG, mmHg (mean ± SD)	18.8 ± 9.0	27.8 ± 8.4		18.3 ± 6.7	23.0 ± 9.2		21.2 ± 3.9	25.4 ± 14.6	
Mean ATPG, mmHg (mean ± SD)	9.5 ± 4.2	15.3 ± 4.6		9.0 ± 3.1	12.7 ± 4.5		11.8 ± 2.1	13.9 ± 9.2	
LVEDD, cm (mean ± SD)	5.0 ± 0.9	5.2 ± 0.3		4.9 ± 0.7	4.9 ± 0.5		4.7 ± 0.7	4.7 ± 0.8	
IVST, cm (mean ± SD)	1.3 ± 0.2	1.4 ± 0.2		1.3 ± 0.2	1.5 ± 0.3		1.4 ± 0.3	1.3 ± 0.3	
PWT, cm (mean ± SD)	0.9 ± 0.1	1.2 ± 0.1	**	1.0 ± 0.1	1.1 ± 0.2	^0.06^	1.1 ± 0.1	1.0 ± 0.2	
RWT (mean ± SD)	0.38 ± 0.09	0.45 ± 0.06		0.41 ± 0.09	0.48 ± 0.10		0.48 ± 0.08	0.45 ± 0.16	
LVMi, g/m^2^ (mean ± SD)	120.2 ± 23.5	157.1 ± 21.5	^0.06^	114.8 ± 23.3	149.5 ± 35.5	^0.05^	119.9 ± 36.1	117.8 ± 18.2	
∆LVM, % (mean ± SD)	30.8 ± 11.6	− 8.7 ± 5.1	^§^	28.5 ± 9.4	− 13.8 ± 16.5	^§^	34.3 ± 14.7	− 16.0 ± 14.0	^§^

^*^p < 0.05 ** *p* < 0.01 *** *p* < 0.001 (unpaired two-tailed t-test); # *p* < 0.05 (Fisher’s exact two-sided test); § Independent variable

^a^Information for this variable is missing for one patient

*ACEi* angiotensin-converting enzyme inhibitors, *ATPG* aortic transvalvular pressure gradient, *AT2Ri* angiotensin II receptor inhibitors, *AVAi* aortic valve area, indexed to body surface area, *BMI* body mass index, *CAD* coronary artery disease, *COPD* chronic obstructive pulmonary disease, *EOAi* effective orifice area, indexed to body surface area, *IVST* interventricular septal thickness, *LVEDD* left ventricle end-diastolic dimension, *LVMi* left ventricle mass, indexed to body surface area, *MRA* mineralocorticoid receptor antagonists, *Peak Ao* peak aortic valve velocity, *PWT* posterior wall thickness, *RWT* relative wall thickness, ∆*LVM* left ventricle mass regression

### The myocardial proteome profile in patients with complete and incomplete reverse remodelling

**Fig. 1:**
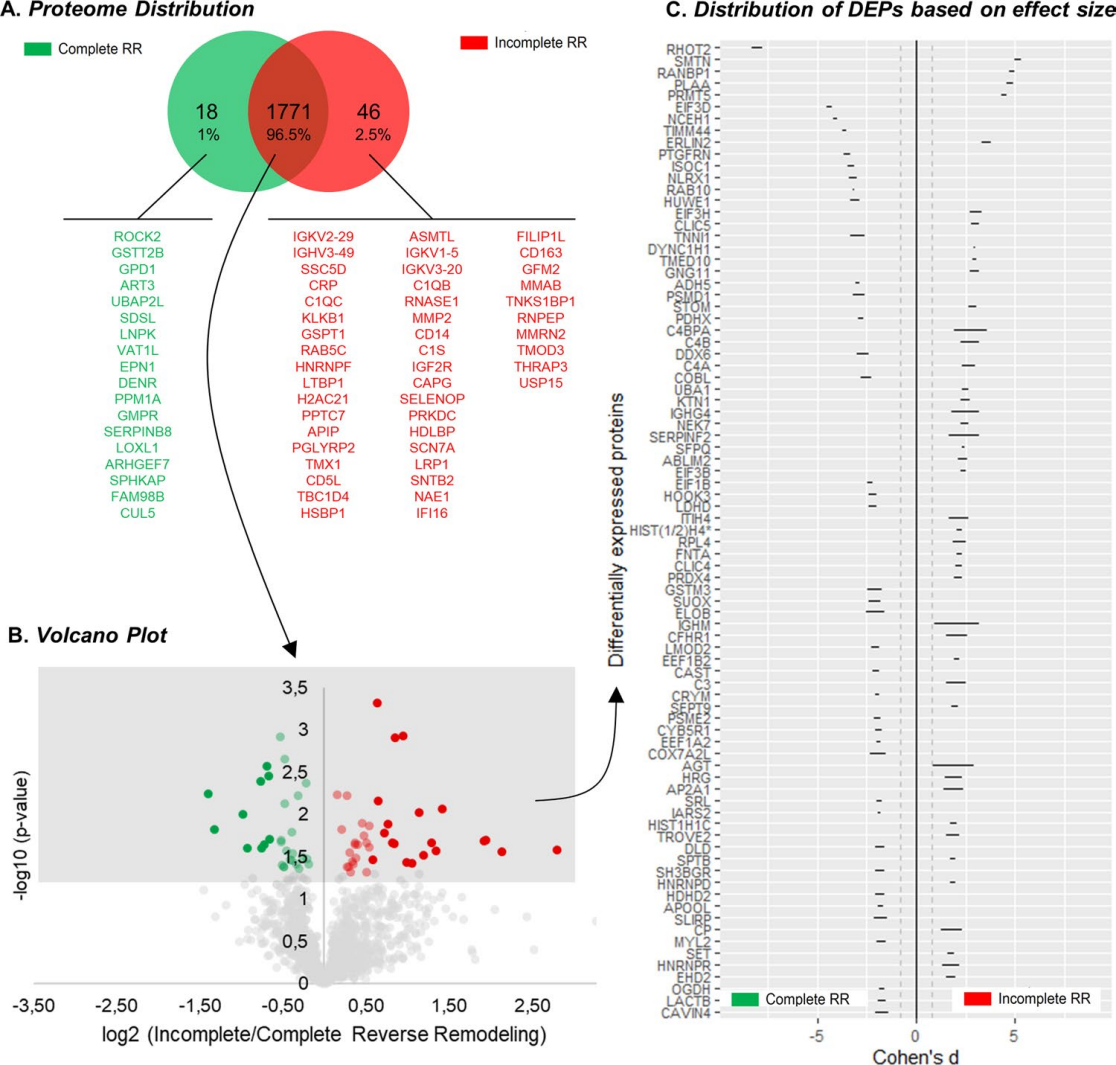
Characterisation of the myocardial proteome in AVS patients with complete (*n* = 4, green) or incomplete (*n* = 4, red) RR. Proteins are identified by their gene name. **A** Venn chart showing the distribution of the proteins identified in both groups. Exclusive proteins are listed. **B** Volcano plot representing DEPs (coloured dots), identified by *t*-test. Darker green and red dots depict, respectively, proteins at least 1.5-fold lower or higher in incomplete RR. **C** Ranking of the proteins according to the effect size (95% confidence interval). Vertical dashed lines mark the large effect size threshold (Cohen’s *d* > 0.8)

From over 1,800 proteins quantified in human myocardium, 1% were identified exclusively in cRR, including the glutathione S-transferase theta-2B (GSTT2B) or the cytoplasmic glycerol-3-phosphate dehydrogenase (GPD1), and 2.5% were only identified in iRR patients, remarkably the C-reactive protein (CRP) and complement components such as C1QC, C1QB or C1S (Fig. 1A). 83 proteins were found dysregulated between cRR and iRR (Fig. 1B and Table 2). All these proteins presented a large effect size (Cohen’s *d* > 0.8, Table 2), and their variation in RR was consistent (*vide* confidence intervals, Fig. 1C). 39 proteins were downregulated in iRR (green dots, Fig. 1B), and the remaining 44 were upregulated in iRR (red dots, Fig. 1B). Moreover, 15 downregulated and 25 upregulated proteins changed > 1.5-fold. For instance, the slow-twitch skeletal muscle isoform of troponin I (ssTnI) and proteostasis-associated proteins, such as the non-ATPase regulatory subunit 1 of the 26S proteasome (PSMD1) and the E3 ubiquitin ligase HUWE1 were found downregulated. In turn, angiotensinogen and complement system elements (e.g. complement C3, C4-A, C4-B and factor H-related protein 1) were upregulated in patients later presenting iRR.

**Table 2:** Differentially expressed proteins in incomplete reverse remodelling. Proteins are sorted in descending order of the fold-change

UniProt ID	Gene name	Full name	# Pep	Seq. Cov. (%)	n (cRR)	n (iRR)	FC	Cohen's d	*p*
P01871	IGHM	Immunoglobulin heavy constant µ	13	34.7	4	4	7.0	2.08	0.026
P04003	C4BPA	C4b-binding protein α chain	5	12.7	3	3	4.4	2.76	0.028
P01019	AGT	Angiotensinogen	6	18.1	4	3	4.2	1.90	0.046
P08697	SERPINF2	α-2-antiplasmin	3	9	4	3	3.8	2.41	0.020
P01861	IGHG4	Immunoglobulin heavy constant γ 4	10	52	3	4	3.8	2.48	0.021
P0C0L5	C4B	Complement C4-B	50	43.2	4	4	2.7	2.72	0.009
Q03591	CFHR1	Complement factor H-related protein 1	9	32.7	4	4	2.5	2.07	0.027
Q14624	ITIH4	Inter-α-trypsin inhibitor heavy chain H4	18	24.1	4	4	2.5	2.18	0.022
P01024	C3	Complement C3	82	55.7	4	4	2.3	2.00	0.030
P0C0L4	C4A	Complement C4-A	50	43.2	4	4	2.2	2.65	0.009
P00450	CP	Ceruloplasmin	29	42.3	4	4	2.2	1.78	0.046
O95782	AP2A1	AP-2 complex sub. α-1	3	5.7	4	4	2.1	1.88	0.038
P04196	HRG	Histidine-rich glycoprotein	11	27.6	4	4	2.0	1.89	0.037
P53814	SMTN	Smoothelin	4	6.3	3	4	1.9	5.11	0.001
O43390	HNRNPR	Heterogeneous nuclear ribonucleoprotein R	5	11.4	4	4	1.8	1.75	0.048
Q9Y263	PLAA	Phospholipase A-2-activating protein	3	6.3	3	4	1.8	4.72	0.001
P36578	RPL4	60S ribosomal protein L4	6	25.1	4	4	1.8	2.16	0.022
O15372	EIF3H	Eukaryotic translation initiation factor 3 sub. H	3	14.5	3	3	1.8	2.99	0.022
Q86UP2	KTN1	Kinectin	11	10.1	4	4	1.7	2.48	0.013
P10155	RO60	60 kDa SS-A/Ro ribonucleoprotein	2	5	4	4	1.7	1.85	0.040
P61952	GNG11	Guanine nucleotide-binding protein G(I)/G(S)/G(O) sub. γ-11	2	27.4	4	3	1.7	2.94	0.016
O94905	ERLIN2	Erlin-2	3	13.9	2	3	1.7	3.56	0.041
P27105	STOM	Erythrocyte band 7 integral membrane protein	5	30.2	4	4	1.6	2.85	0.007
P43487	RANBP1	Ran-specific GTPase-activating protein	3	14.9	4	4	1.6	4.83	0.000
Q6H8Q1	ABLIM2	Actin-binding LIM protein 2	4	9	4	3	1.5	2.38	0.034
Q8TDX7	NEK7	Serine/threonine-protein kinase Nek7	3	13.9	4	4	1.5	2.44	0.014
Q13162	PRDX4	Peroxiredoxin-4	8	32.1	4	4	1.5	2.11	0.024
Q9NZA1	CLIC5	Chloride intracellular channel protein 5	5	19	3	3	1.4	2.99	0.022
Q9NZN4	EHD2	EH domain-containing protein 2	18	49.9	4	4	1.4	1.75	0.048
O14744	PRMT5	Protein arginine N-methyltransferase 5	2	6.9	3	2	1.4	4.41	0.018
P22314	UBA1	Ubiquitin-like modifier-activating enzyme 1	26	40.8	4	4	1.4	2.48	0.013
Q9Y696	CLIC4	Chloride intracellular channel protein 4	14	79.8	4	4	1.3	2.15	0.023
Q9UHD8	SEPTIN9	Septin-9	5	17.4	4	4	1.3	1.96	0.032
P49354	FNTA	Protein farnesyltransferase/geranylgeranyltransferase type-1 sub. α	2	7.4	4	4	1.3	2.16	0.022
P62805	HIST*H#^a)^	Histone H4	8	56.3	4	4	1.3	2.18	0.022
P16403	HIST1H1C	Histone H1.2	8	31.9	4	4	1.3	1.86	0.039
P55884	EIF3B	Eukaryotic translation initiation factor 3 sub. B	3	3.8	3	4	1.3	2.35	0.036
Q01105	SET	Protein SET	3	17.2	4	4	1.2	1.75	0.048
P24534	EEF1B2	Elongation factor 1-β	7	44	4	4	1.2	2.03	0.028
Q14103	HNRNPD	Heterogeneous nuclear ribonucleoprotein D0	8	24.5	4	4	1.2	1.83	0.041
P49755	TMED10	Transmembrane emp24 domain-containing protein 10	4	28.8	4	4	1.2	2.94	0.006
P11277	SPTB	Spectrin β chain, erythrocytic	36	22.8	4	4	1.2	1.83	0.041
P23246	SFPQ	Splicing factor, proline- and glutamine-rich	8	15.8	4	4	1.2	2.38	0.015
Q14204	DYNC1H1	Cytoplasmic dynein 1 heavy chain 1	72	20.7	4	4	1.1	2.96	0.006
Q9NSE4	IARS2	Isoleucine–tRNA ligase, mitochondrial	17	23.2	4	4	− 1.1	1.86	0.039
Q05639	EEF1A2	Elongation factor 1-α 2	23	65.2	4	4	− 1.2	1.92	0.035
P61026	RAB10	Ras-related protein Rab-10	5	24.5	4	4	− 1.2	3.16	0.004
Q02218	OGDH	2-oxoglutarate dehydrogenase, mitochondrial	60	60.5	4	4	− 1.2	1.74	0.049
Q14894	CRYM	Ketimine reductase µ-crystallin	17	69.4	4	4	− 1.2	1.97	0.032
Q6UXV4	APOOL	MICOS complex sub. MIC27	10	57.1	4	4	− 1.2	1.81	0.043
P11766	ADH5	Alcohol dehydrogenase class-3	15	50.3	4	4	− 1.2	2.94	0.006
Q86TD4	SRL	Sarcalumenin	28	28.9	4	4	− 1.3	1.86	0.039
O15371	EIF3D	Eukaryotic translation initiation factor 3 sub. D	3	7.7	2	2	− 1.3	4.39	0.048
P20810	CAST	Calpastatin	16	43.5	4	4	− 1.3	2.03	0.029
O60739	EIF1B	Eukaryotic translation initiation factor 1b	5	54	4	4	− 1.3	2.34	0.016
Q9UHQ9	CYB5R1	NADH-cytochrome b5 reductase 1	15	60.7	4	4	− 1.3	1.93	0.035
Q9UL46	PSME2	Proteasome activator complex sub. 2	4	24.7	4	4	− 1.3	1.95	0.033
P83111	LACTB	Serine β-lactamase-like protein LACTB, mitochondrial	8	21.4	4	4	− 1.3	1.74	0.049
Q6P5Q4	LMOD2	Leiomodin-2	9	22.9	4	4	− 1.4	2.06	0.027
O43615	TIMM44	Mitochondrial import inner membrane translocase sub. TIM44	6	18.8	4	4	− 1.4	3.62	0.002
P10916	MYL2	Myosin regulatory light chain 2, ventricular/cardiac muscle isoform	30	93.4	4	4	− 1.4	1.77	0.046
O00330	PDHX	Pyruvate dehydrogenase protein X component, mitochondrial	15	36.9	4	4	− 1.4	2.80	0.007
P55822	SH3BGR	SH3 domain-binding glutamic acid-rich protein	8	38.9	4	4	− 1.4	1.83	0.042
Q9H0R4	HDHD2	Haloacid dehalogenase-like hydrolase domain-containing protein 2	2	13.1	4	4	− 1.4	1.83	0.042
P09622	DLD	Dihydrolipoyl dehydrogenase, mitochondrial	26	67.6	4	4	− 1.4	1.85	0.040
Q86WU2	LDHD	Probable D-lactate dehydrogenase, mitochondrial	15	57	4	4	− 1.4	2.19	0.021
Q86VS8	HOOK3	Protein Hook homolog 3	3	4.9	4	4	− 1.4	2.23	0.020
Q6PIU2	NCEH1	Neutral cholesterol ester hydrolase 1	8	30.6	4	4	− 1.4	4.07	0.001
Q7Z6Z7	HUWE1	E3 ubiquitin-protein ligase HUWE1	4	1.5	3	3	− 1.6	3.07	0.020
Q96CN7	ISOC1	Isochorismatase domain-containing protein 1	5	27.9	4	4	− 1.6	3.28	0.004
Q5BKX8	CAVIN4	Caveolae-associated protein 4	5	14.6	4	4	− 1.6	1.74	0.049
Q9P2B2	PTGFRN	Prostaglandin F2 receptor negative regulator	4	5.9	4	4	− 1.6	3.47	0.003
P26196	DDX6	Probable ATP-dependent RNA helicase DDX6	3	9.5	3	3	− 1.6	2.70	0.030
O75128	COBL	Protein cordon-bleu	2	2.2	4	3	− 1.6	2.54	0.022
Q9GZT3	SLIRP	SRA stem-loop-interacting RNA-binding protein, mitochondrial	4	45.9	4	4	− 1.7	1.79	0.044
P51687	SUOX	Sulphite oxidase, mitochondrial	5	18.3	4	4	− 1.7	2.10	0.025
Q86UT6	NLRX1	NLR family member X1	5	5.7	4	4	− 1.7	3.19	0.004
O14548	COX7A2L	Cytochrome c oxidase sub. 7A-related protein, mitochondrial	2	40.4	4	4	− 1.9	1.91	0.036
P21266	GSTM3	Glutathione S-transferase µ 3	9	51.6	4	4	− 1.9	2.11	0.025
Q99460	PSMD1	26S proteasome non-ATPase regulatory sub. 1	3	4.5	4	3	− 2.0	2.91	0.010
Q15370	ELOB	Elongin-B	3	39	4	4	− 2.3	2.09	0.025
Q8IXI1	RHOT2	Mitochondrial Rho GTPase 2	3	7.4	2	2	− 2.5	8.06	0.015
P19237	TNNI1	Troponin I, slow skeletal muscle	10	47.1	4	4	− 2.6	2.96	0.006

*FC* fold-change, *Pep*. Peptides, *Seq. Cov.* sequence coverage

#### Gene ontology enrichment analysis

**Fig. 2:**
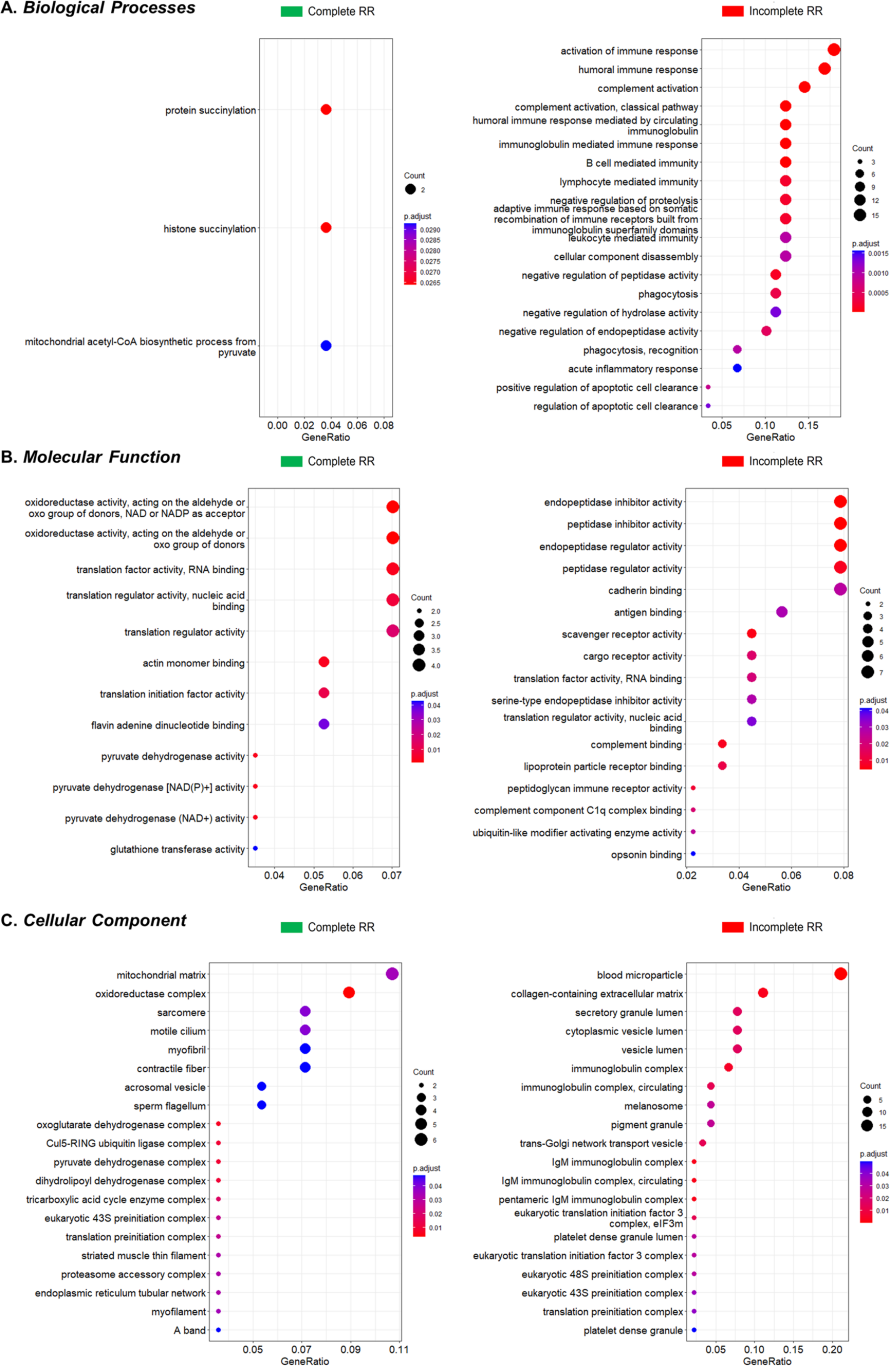
Gene ontology enrichment analysis of the DEPs. The top 20 enriched biological processes (**A**), molecular functions (**B**), and cellular components (**C**) are shown for complete and incomplete RR. The terms are ranked according to the gene ratio and the FDR-adjusted *p*-value (hypergeometric test). The dot size is proportional to gene count and its colour is scaled to the significance level

**Fig. 3:**
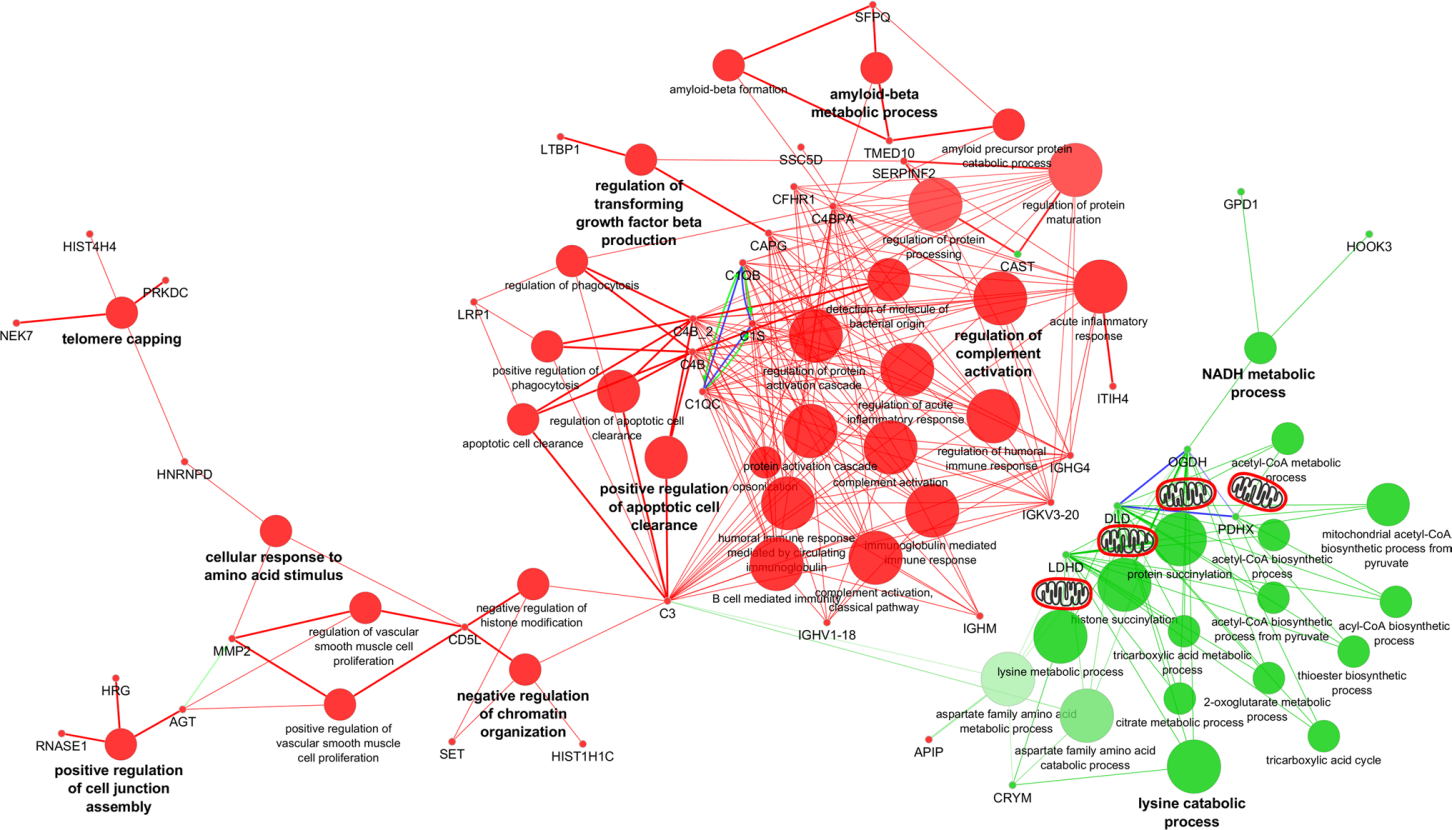
Network of the dysregulated biological processes between complete (green) and incomplete (red) RR, after ClueGO analysis. Proteins are identified by their gene name. Protein–protein interactions were added with CluePedia to depict functional nodules. One such nodule is a hub of four mitochondrial proteins upregulated in complete RR (*vide* mitochondria icons)

The enriched biological processes/pathways, molecular functions and cellular components are shown in Fig. 2 and Table S2. Concerning BP, the GOEA suggests a marked activation of the immune system, including humoral and cell-mediated responses, activation of acute inflammatory reactions and the complement system in patients presenting with iRR. In turn, patients with a cRR seem to have increased protein succinylation and higher mitochondrial activity, supported by increased acetyl-CoA synthesis from pyruvate (Fig. 2A). Indeed, GOEA globally suggests a higher mitochondrial activity/content in these patients, as indicated by an enrichment of the MF oxidoreductase activity, flavin adenine dinucleotide binding or pyruvate dehydrogenase activity (Fig. 2B), or by the most relevant protein localisation in cRR being the mitochondrial matrix (Fig. 2C). As for iRR, MF terms enrichment, such as antigen binding or complement binding, reinforces the activation of the immune-inflammatory responses in the myocardium (Fig. 2B). Naturally, many proteins participating in these pathways are secreted from injured cells, explaining an enrichment of blood microparticle, collagen-containing ECM or vesicle lumen localisations (Fig. 2C). A deeper GOEA analysis further supports a downregulation of NADH metabolism and lysine catabolism and upregulation of complement system activation, TGF-β production regulation and apoptotic cell clearance in patients with iRR. A hub of interacting mitochondrial proteins was associated with patients with cRR (Fig. 3).

#### Metabolic profiling

**Fig. 4:**
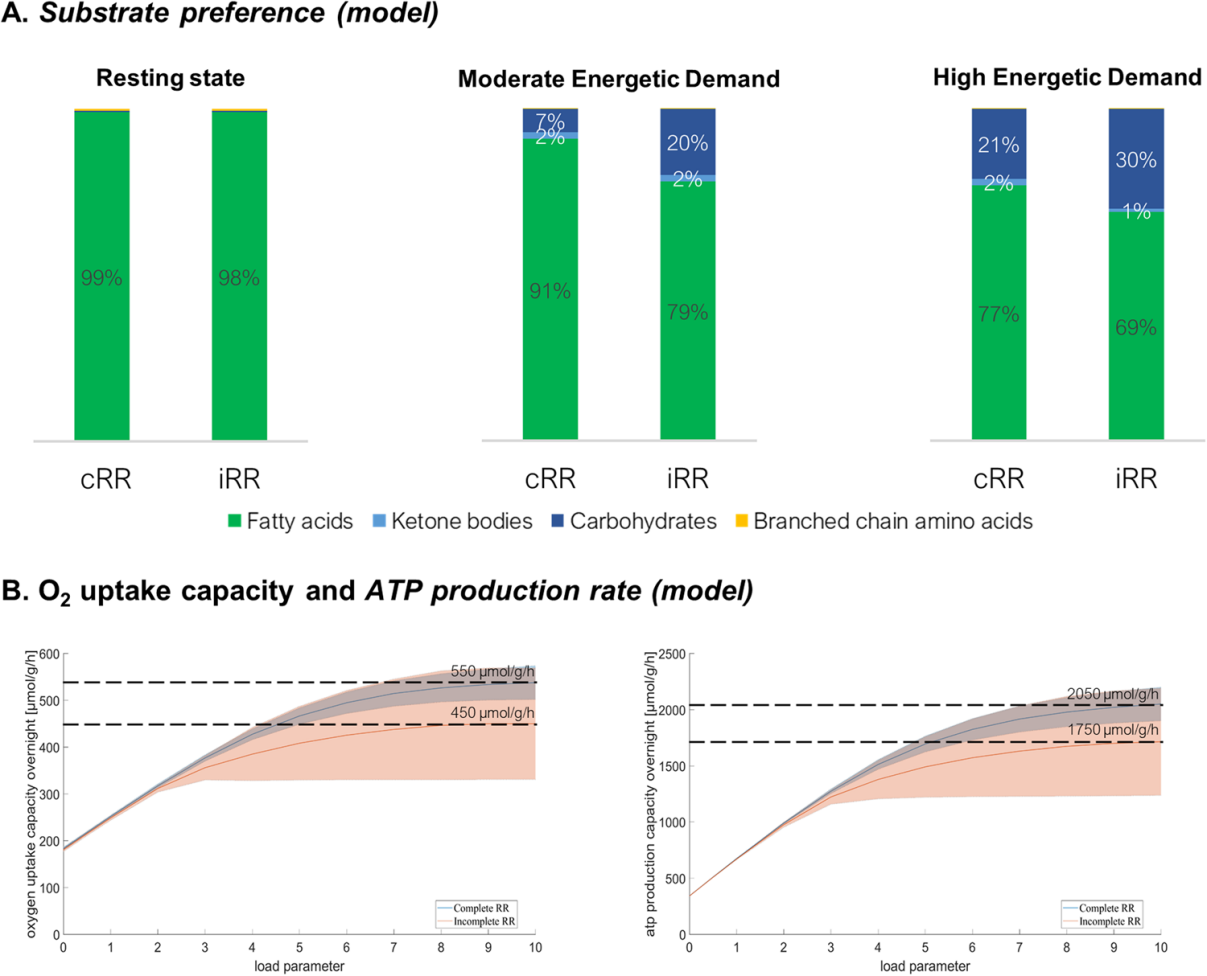
Metabolic profiling with Quantitative System Metabolism^™^. **A** Prediction of substrate preference according to different energetic demands. In a resting state, fatty acids (green) are predicted as the preferred energy source for all patients. As the energetic demand rises, so does the utilisation of carbohydrates (dark blue). However, reliance on fatty acids as an energetic substrate is better preserved in patients with complete RR (77% vs 69% under high energetic stress). Ketone bodies (light blue) and branched-chain amino acids (yellow) are used in residual amounts. **B** Prediction of oxygen uptake and ATP production with increasing workload. Maximal oxygen consumption and ATP production rate plateau at a lower level in incomplete RR patients

One of the main differences suggested by GOEA is better preservation of the mitochondrial metabolism in cRR. Therefore, we used a kinetic model of cardiac metabolism to further explore these differences. As expected, fatty acids are the preferred energetic substrate, and carbohydrates become increasingly utilised as the energetic demand increases (Fig. 4A). However, the reliance of iRR patients on fatty acids is lower under high energetic stress (69% versus 77%), compensated by increased utilisation of carbohydrates (30% versus 21%). This model suggests that, under high workload, the switch to a less aerobic metabolism is more pronounced in iRR, as explained by a lower maximal oxygen uptake and ATP production capacity (Fig. 4B).

### The myocardial phosphoproteome profile in patients with complete and incomplete reverse remodelling

**Fig. 5:**
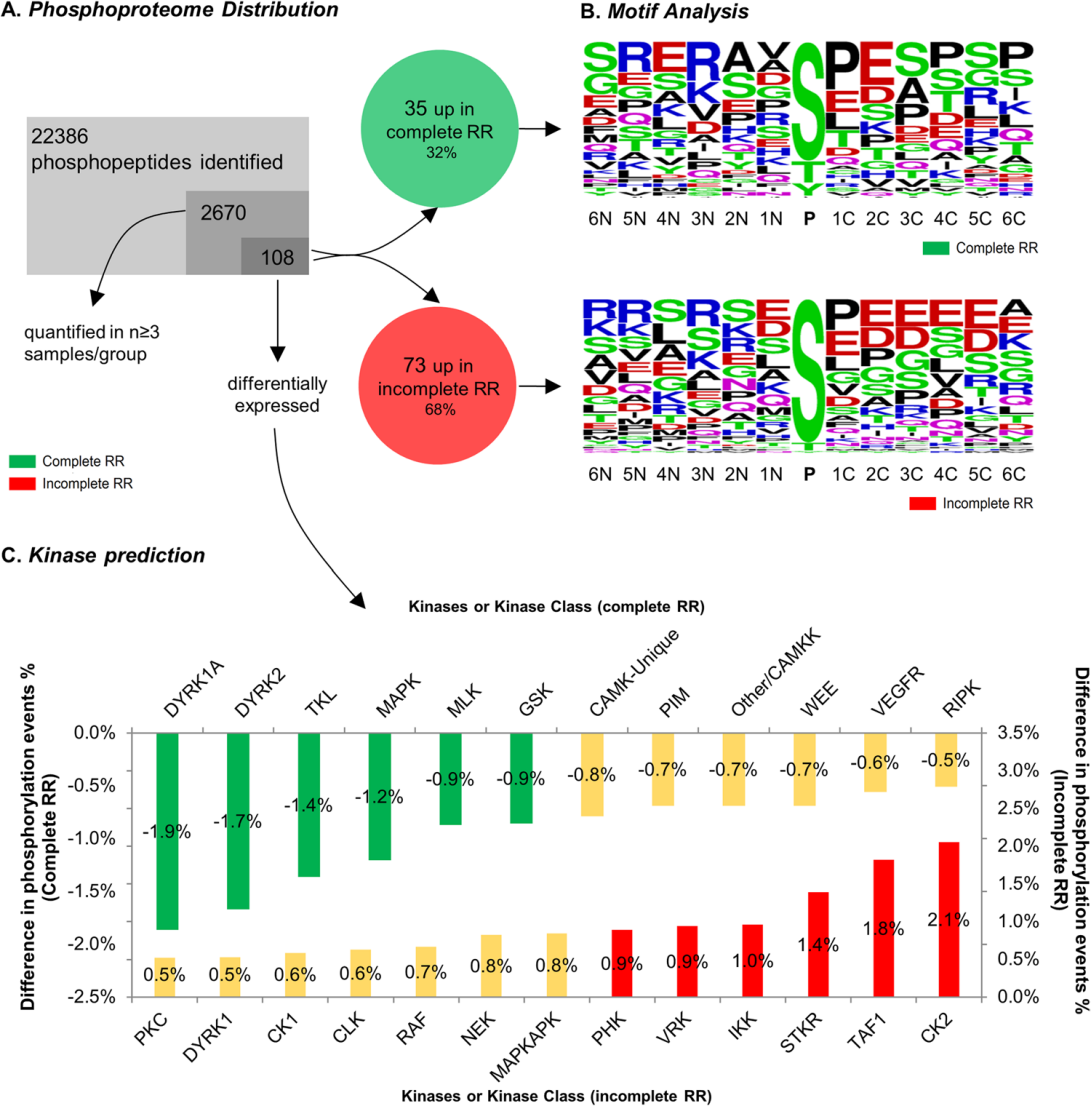
Characterisation of the myocardial phosphoproteome in AVS patients with complete (*n* = 4, green) or incomplete (*n* = 4, red) RR. **A** Representation of the identified and differentially expressed phosphopeptides. **B** Motif analysis of the phosphosites’ neighbouring sequence in complete (top) and incomplete (bottom) RR. Amino acids are depicted by the single letter code. **C** Kinase rank. Predicted kinases are sorted according to the percentage of associated phosphorylation events. Only kinases with a difference |cRR-iRR|> 0.5% in phosphorylation events are shown (Table S3 provides the complete list). Kinases (or their families) in the first and tenth percentiles were associated with incomplete (red) and complete RR (green), respectively

Aiming to disclose the most dysregulated kinases between patients with diverging RR phenotypes, we have analysed the phosphoproteome of the same myocardial biopsies. Over 22,000 phosphopeptides were identified, and 2,600 were quantified in ≥ 3 subjects per group. 108 phosphopeptides were found dysregulated (73 upregulated and 35 downregulated) in the myocardium of patients with iRR (Fig. 5A and Table 3).

**Table 3:** Differentially expressed phosphopeptides in incomplete reverse remodelling. Phosphopeptides are sorted in descending order of the fold-change

UniProt ID	Gene name	Protein name	Phosphopeptide	Seq pos	AA	FC	*p*
P41208	CETN2	Centrin-2	RMpSPKPELTEEQK	20	S	3.7	0.003
Q8IYB7	DIS3L2	DIS3-like exonuclease 2	RPGTQGHLGPEKEEEEpSDGEPEDSSTS	875	S	3.6	0.004
Q13459	MYO9B	Unconventional myosin-IXb	VQEKPDpSPGGSTQIQR	1290	S	3.5	0.003
Q15842	KCNJ8	ATP-sensitive inward rectifier potassium channel 8	pSIIPEEYVLAR	6	S	3.4	0.036
P02765	AHSG	α-2-HS-glycoprotein	CDSSPDpSAEDVRK	138	S	3.3	0.015
Q7Z5L9	IRF2BP2	Interferon regulatory factor 2-binding protein 2	RKPpSPEPEGEVGPPK	360	S	3.3	0.022
P46976	GYG1	Glycogenin-1	ERWEQGQADYMGADpSFDNIK	337	S	3.1	0.028
P08559	PDHA1	Pyruvate dehydrogenase E1 component sub. α, somatic form, mitochondrial	VLSGApSQKPASR	16	S	3.1	0.043
O94811	TPPP	Tubulin polymerisation-promoting protein	AANRTPPKpSPGDPSK	18	S	2.8	0.010
O94811	TPPP	Tubulin polymerisation-promoting protein	AANRpTPPKSPGDPSK	14	T	2.8	0.010
Q9UHD8	SEPT9	Septin-9	pSVQPTSEERIPK	327	S	2.8	0.007
Q8WZ42	TTN	Titin	RLpSDHSVEPGK	6920	S	2.8	0.049
O95359	TACC2	Transforming acidic coiled-coil-containing protein 2	DLSRSpSDSEEAFETPESTTPVK	1947	S	2.8	0.028
Q13557	CAMK2D	Calcium/calmodulin-dependent protein kinase type II sub. δ	KPDGVKEpSTESSNTTIEDEDVK	330	S	2.8	0.004
P08670	VIM	Vimentin	QVQpSLTCEVDALK	325	S	2.5	0.025
O00192	ARVCF	Armadillo repeat protein deleted in velo-cardio-facial syndrome	DGEMDRNFDpTLDLPK	642	T	2.4	0.031
O15234	CASC3	Protein CASC3	STVTGERQpSGDGQESTEPVENK	148	S	2.2	0.024
Q8IYB3	SRRM1	Serine/arginine repetitive matrix protein 1	KVELpSESEEDKGGK	463	S	2.2	0.047
Q14151	SAFB2	Scaffold attachment factor B2	VVTNARpSPGAR	444	S	2.2	0.040
Q5UIP0	RIF1	Telomere-associated protein RIF1	SNEpSVDIQDQEEK	1579	S	2.2	0.003
Q01082	SPTBN1	Spectrin β chain, non-erythrocytic 1	ESpSPIPSPTSDRK	2165	S	2.1	0.039
P29590	PML	Protein PML	VIKMEpSEEGK	493	S	2.1	0.020
Q9UHB6	LIMA1	LIM domain and actin-binding protein 1	QQpSPQEPK	698	S	2.1	0.016
Q96CB8	INTS12	Integrator complex sub. 12	SVpSCDNVSK	378	S	2.1	0.017
P61981	YWHAG	14–3-3 protein γ	VISSIEQKTpSADGNEK	71	S	2.0	0.015
P02545	LMNA	Prelamin-A/C	ASSHSpSQTQGGGSVTK	407	S	2.0	0.022
Q09666	AHNAK	Neuroblast differentiation-associated protein AHNAK	MpSLPDVDLDLK	1068	S	2.0	0.010
Q13557	CAMK2D	Calcium/calmodulin-dependent protein kinase type II sub. δ	ESTEpSSNTTIEDEDVK	333	S	2.0	0.008
Q99759	MAP3K3	Mitogen-activated protein kinase kinase kinase 3	AQpSFPDNRQEYSDRETQLYDK	250	S	1.9	0.021
P13637	ATP1A3	Sodium/potassium-transporting ATPase sub. α-3	YNTDCVQGLTHpSK	56	S	1.9	0.029
Q13428	TCOF1	Treacle protein	AALAPAKEpSPRK	906	S	1.9	0.040
O15021	MAST4	Microtubule-associated serine/threonine-protein kinase 4	SQALGQpSAPSLTASLK	206	S	1.9	0.043
O15021	MAST4	Microtubule-associated serine/threonine-protein kinase 4	SQALGQSAPSLTApSLK	213	S	1.9	0.043
Q99549	MPHOSPH8	M-phase phosphoprotein 8	GAEAFGDpSEEDGEDVFEVEK	51	S	1.9	0.010
P43003	SLC1A3	Excitatory amino acid transporter 1	NRDVEMGNpSVIEENEMK	512	S	1.8	0.042
Q14157	UBAP2L	Ubiquitin-associated protein 2-like	STSAPQMpSPGSSDNQSSSPQPAQQK	467	S	1.8	0.006
Q9GZR7	DDX24	ATP-dependent RNA helicase DDX24	AQAVpSEEEEEEEGK	82	S	1.8	0.022
Q9UGV2	NDRG3	Protein NDRG3	SRTHSTSSpSLGSGESPFSR	335	S	1.8	0.026
P23497	SP100	Nuclear autoantigen Sp-100	KRVIGQDHDFpSESSEEEAPAEASSGALR	407	S	1.8	0.021
Q3KQU3	MAP7D1	MAP7 domain-containing protein 1	RSpSQPSPTAVPASDSPPTK	113	S	1.8	0.033
Q6GYQ0	RALGAPA1	Ral GTPase-activating protein sub. α-1	pTVDIDDAQILPR	754	T	1.8	0.042
Q9Y2X7	GIT1	ARF GTPase-activating protein GIT1	HGpSGADSDYENTQSGDPLLGLEGK	592	S	1.8	0.029
Q9Y2X7	GIT1	ARF GTPase-activating protein GIT1	LSRHGSGADSDpYENTQSGDPLLGLEGK	598	Y	1.8	0.029
Q9UQ35	SRRM2	Serine/arginine repetitive matrix protein 2	THTTALAGRSPpSPASGR	297	S	1.8	0.042
Q9UQ35	SRRM2	Serine/arginine repetitive matrix protein 2	THTTALAGRSPSPApSGRR	300	S	1.8	0.042
Q9NYL9	TMOD3	Tropomodulin-3	DLDEDELLGNLpSETELK	25	S	1.8	0.039
Q29RF7	PDS5A	Sister chromatid cohesion protein PDS5 homolog A	RAAVGQEpSPGGLEAGNAK	1305	S	1.8	0.005
Q92609	TBC1D5	TBC1 domain family member 5	SEpSMPVQLNK	522	S	1.7	0.050
Q9UIG0	BAZ1B	Tyrosine-protein kinase BAZ1B	FPDRLAEDEGDpSEPEAVGQSR	1468	S	1.7	0.045
Q14195	DPYSL3	Dihydropyrimidinase-related protein 3	NLHQSGFSLpSGTQVDEGVR	541	S	1.7	0.027
Q9UKJ3	GPATCH8	G patch domain-containing protein 8	SQpSPHYFR	1035	S	1.7	0.034
O60271	SPAG9	C-Jun-amino-terminal kinase-interacting protein 4	SASQSpSLDKLDQELK	733	S	1.7	0.047
P02545	LMNA	Prelamin-A/C	pSNEDQSMGNWQIK	458	S	1.7	0.002
O14974	PPP1R12A	Protein phosphatase 1 regulatory sub. 12A	SApSSPRLSSSLDNK	472	S	1.7	0.003
Q9Y5K6	CD2AP	CD2-associated protein	pSVDFDSLTVR	458	S	1.7	0.035
O75475	PSIP1	PC4 and SFRS1-interacting protein	EDTDHEEKApSNEDVTK	129	S	1.7	0.046
Q8IYB3	SRRM1	Serine/arginine repetitive matrix protein 1	KVELpSESEEDKGGK	463	S	1.6	0.016
Q8IYB3	SRRM1	Serine/arginine repetitive matrix protein 1	KVELSEpSEEDKGGK	465	S	1.6	0.016
P62328	TMSB4X	Thymosin β-4	pSDKPDMAEIEKFDK	2	S	1.6	0.014
O95453	PARN	Poly(A)-specific ribonuclease PARN	ELpSPAGSISK	619	S	1.6	0.000
Q8NE71	ABCF1	ATP-binding cassette sub-family F member 1	KLpSVPTpDEEDEVPAPKPR	105	S	1.5	0.020
Q13557	CAMK2D	Calcium/calmodulin-dependent protein kinase type II sub. δ	ESTESSNTpTIEDEDVK	337	T	1.5	0.037
Q01813	PFKP	ATP-dependent 6-phosphofructokinase, platelet type	GRpSFAGNLNTYK	386	S	1.5	0.005
Q9H0B6	KLC2	Kinesin light chain 2	TLSSpSSMDLSR	610	S	1.5	0.047
P16284	PECAM1	Platelet endothelial cell adhesion molecule	YSRTEGpSLDGT	734	S	1.4	0.013
Q92597	NDRG1	Protein NDRG1	TApSGSSVTSLDGTR	330	S	1.4	0.029
Q9H246	C1orf21	Uncharacterised protein C1orf21	GRDYCpSEEEDIT	115	S	1.4	0.021
Q7Z6E9	RBBP6	E3 ubiquitin-protein ligase RBBP6	WDKDDFEpSEEEDVK	1328	S	1.4	0.039
O75351	VPS4B	Vacuolar protein sorting-associated protein 4B	GNDpSDGEGESDDPEKK	102	S	1.4	0.021
O95070	YIF1A	Protein YIF1A	AYHpSGYGAHGSK	5	S	1.3	0.049
O95070	YIF1A	Protein YIF1A	AYHSGYGAHGpSK	12	S	1.3	0.049
Q86VM9	ZC3H18	Zinc finger CCCH domain-containing protein 18	LGVSVpSPSR	534	S	1.2	0.049
P08648	ITGA5	Integrin α-5	LLESSLSpSSEGEEPVEYK	127	S	1.2	0.039
P30566	ADSL	Adenylosuccinate lyase	AAGGDHGpSPDSYRSPLASR	9	S	− 1.4	0.032
Q09666	AHNAK	Neuroblast differentiation-associated protein AHNAK	VDVEVPDVpSLEGPEGK	1298	S	− 1.5	0.045
Q9HBL0	TNS1	Tensin-1	SGpSLGQPSPSAQR	1119	S	− 1.6	0.047
Q9HBL0	TNS1	Tensin-1	SGSLGQPpSPSAQR	1124	S	− 1.6	0.047
Q8WX93	PALLD	Palladin	RAIADSETEDFDpSEK	60	S	− 1.6	0.038
Q4G0J3	LARP7	La-related protein 7	RKRSSpSEDAESLAPR	300	S	− 1.9	0.036
Q6PKG0	LARP1	La-related protein 1	ETESAPGSPRAVpTPVPTK	526	T	− 1.9	0.011
O75151	PHF2	Lysine-specific demethylase PHF2	EDKPKPVRDEYEYVpSDDGELK	681	S	− 1.9	0.040
Q2M3C7	SPHKAP	A-kinase anchor protein SPHKAP	QSpSCESITDEFSR	1121	S	− 1.9	0.041
Q9H1B7	IRF2BPL	Interferon regulatory factor 2-binding protein-like	RKApSPEPPDSAEGALK	547	S	− 1.9	0.007
P12883	MYH7	Myosin-7	NLpTEEMAGLDEIIAK	979	T	− 1.9	0.028
Q9UN36	NDRG2	Protein NDRG2	pSRTASLTSAASVDGNRSR	328	S	− 2.0	0.030
Q9UN36	NDRG2	Protein NDRG2	LSRSRpTASLTSAASVDGNR	330	T	− 2.0	0.030
Q9BXK5	BCL2L13	Bcl-2-like protein 13	pSSPATSLFVELDEEEVK	370	S	− 2.0	0.027
Q8N3K9	CMYA5	Cardiomyopathy-associated protein 5	LVAQpSIEDK	291	S	− 2.0	0.016
Q14247	CTTN	Src substrate cortactin	TQpTPPVSPAPQPTEER	401	T	− 2.1	0.025
P21397	MAOA	Amine oxidase [flavin-containing] A	VLGpSQEALHPVHYEEK	383	S	− 2.1	0.011
Q6PKG0	LARP1	La-related protein 1	EpTESAPGSPRAVTPVPTKTEEVSNLK	515	T	− 2.2	0.005
Q9UBB9	TFIP11	Tuftelin-interacting protein 11	KGAAEEAELEDpSDDEEKPVKQDDFPK	98	S	− 2.2	0.046
P19237	TNNI1	Troponin I, slow skeletal muscle	MFDAAKpSPTSQ	183	S	− 2.3	0.013
Q14247	CTTN	Src substrate cortactin	TQTPPVpSPAPQPTEERLPSSPVYEDAASFK	405	S	− 2.3	0.020
Q96AG3	SLC25A46	Solute carrier family 25 member 46	SFpSTGSDLGHWVTTPPDIPGSR	34	S	− 2.5	0.037
Q15772	SPEG	Striated muscle preferentially expressed protein kinase	AApSVELPQRR	2037	S	− 2.6	0.008
Q6JBY9	RCSD1	CapZ-interacting protein	SSEEVDGQHPAQEEVPEpSPQTSGPEAENR	284	S	− 2.6	0.029
P62070	RRAS2	Ras-related protein R-Ras2	FQEQECPPpSPEPTRK	186	S	− 2.7	0.039
Q99959	PKP2	Plakophilin-2	WGRGTAQYSpSQK	132	S	− 2.7	0.044
Q9UN36	NDRG2	Protein NDRG2	TApSLTSAASVDGNR	332	S	− 3.0	0.001
Q8N3K9	CMYA5	Cardiomyopathy-associated protein 5	GLpSEEVSHPADFK	1752	S	− 3.0	0.048
P10644	PRKAR1A	cAMP-dependent protein kinase type I-α regulatory sub	EDEIpSPPPPNPVVK	83	S	− 3.1	0.027
P40763	STAT3	Signal transducer and activator of transcription 3	YCRPESQEHPEADPGSAAPpYLK	705	Y	− 3.5	0.043
Q99959	PKP2	Plakophilin-2	RLEISPDSSPERAHpYTHSDYQYSQR	161	Y	− 5.8	0.027
Q99959	PKP2	Plakophilin-2	RLEISPDSSPERAHpYTHSDYQYSQR	161	Y	− 8.5	0.027
O14639	ABLIM1	Actin-binding LIM protein 1	MIHRpSTSQGSINSPVYSR	450	S	− 8.6	0.000
O14639	ABLIM1	Actin-binding LIM protein 1	MIHRSTSQGSINpSPVYSR	458	S	− 8.6	0.000
Q14896	MYBPC3	Myosin-binding protein C, cardiac-type	RISDpSHEDTGILDFSSLLK	286	S	− 281.2	0.002

*AA* amino acid residue, *FC* fold-change, *Seq. Pos*. sequence position

#### Motif analysis and kinase prediction

**Fig. 6:**
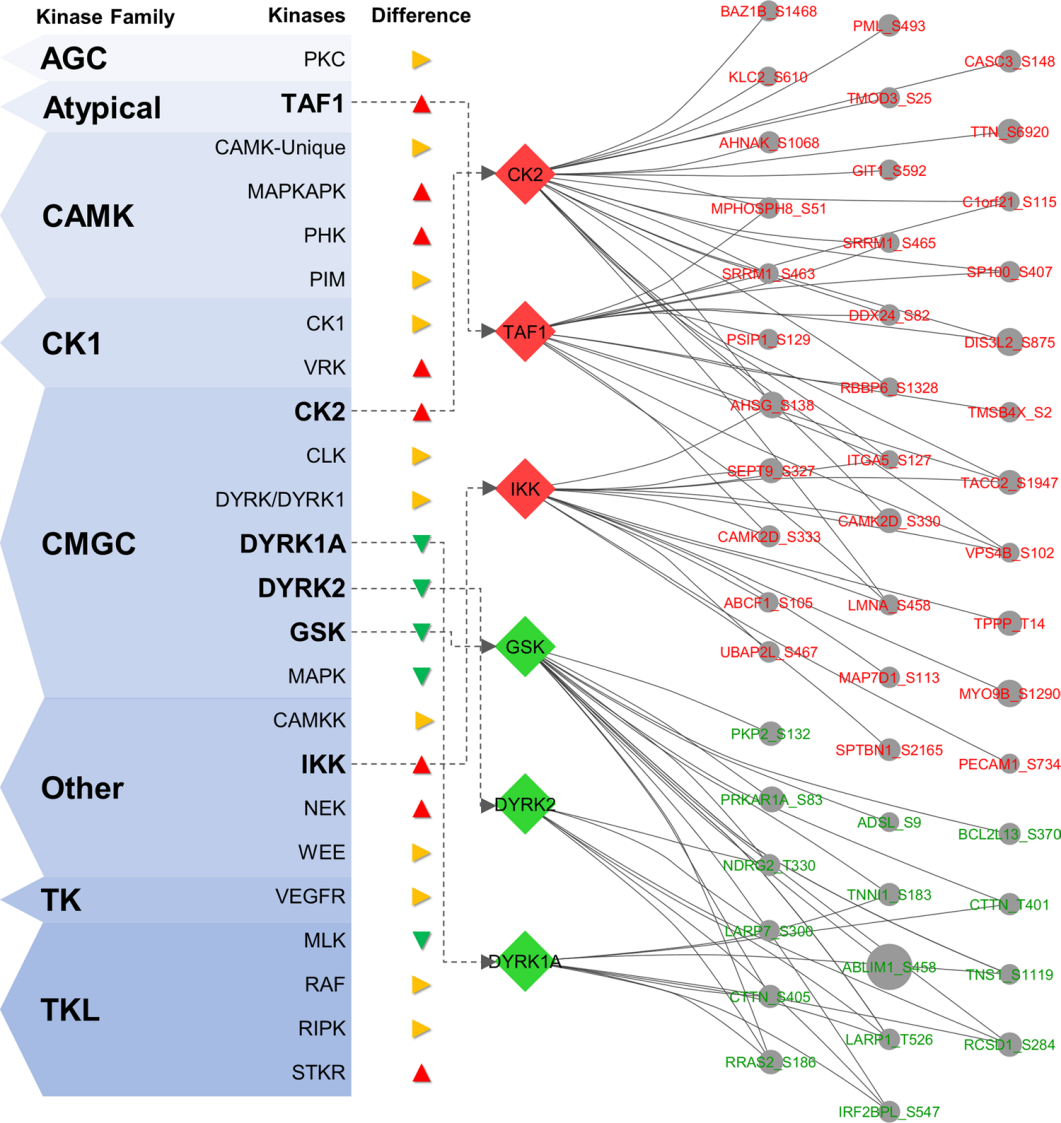
Segregation of the most important kinases, according to the respective kinase class (left panel), highlighting the three most important kinases for complete and incomplete reverse remodelling and the respective phosphosites (on the right). Note that kinase families were left out of the analysis and that IKK was represented instead of STKR family, because the latter comprise kinases phylogenetically more diverse than the former. A high degree of kinase–substrate overlap in both phenotypes could be observed through network analysis (the reader should see the interconnectivity between phosphoproteins, represented by gene name, and the central kinases), suggesting co-regulation or participation in convergent pathways

Some differences in kinase activity between iRR and cRR were suggested after a motif analysis (Fig. 5B). First, in iRR, the dysregulated kinases seem to prefer more acidic residues flanking the target residue at the C-*terminus* side, mainly Glu or Asp in the second, third and fifth positions. Second, in cRR, most kinases seem to prefer nonpolar residues at the first flanking position at the N-*terminus* side (Val and Ala are preferred over Glu or Asp). Third, also in cRR, a higher preference for Pro residues in the C-*terminus* flanking sequence is noticeable, mainly at the first, fourth and sixth positions. These differences were reflected in kinase prediction. Figure 5C illustrates the kinases predicted as the most active in cRR and iRR, according to the difference in the percentage of assigned phosphorylation events. Apart from some families, such as the mitogen-activated protein kinase (MAPK) family, the dual specificity tyrosine phosphorylation-regulated kinase (DYRK) 1A and 2 and the glycogen synthase kinase (GSK, including isoforms α and β) were found to be associated with cRR. In turn, the serine/threonine kinase receptors (STKR), IκB kinase (IKK), vaccinia-related kinase (VRK) families, as well as casein kinase II (CK2), transcription initiation factor TFIID subunit 1 (TAF1) and the phosphorylase kinase (PHK) were deemed the most active kinases in iRR. Figure 6 shows the segregation of the most important kinases according to the respective class and targets. A high degree of kinase–substrate overlap in both phenotypes was observed through network analysis, suggesting co-regulation or participation in close pathways.

### Immunoblot validation and clinical correlations

**Fig. 7:**
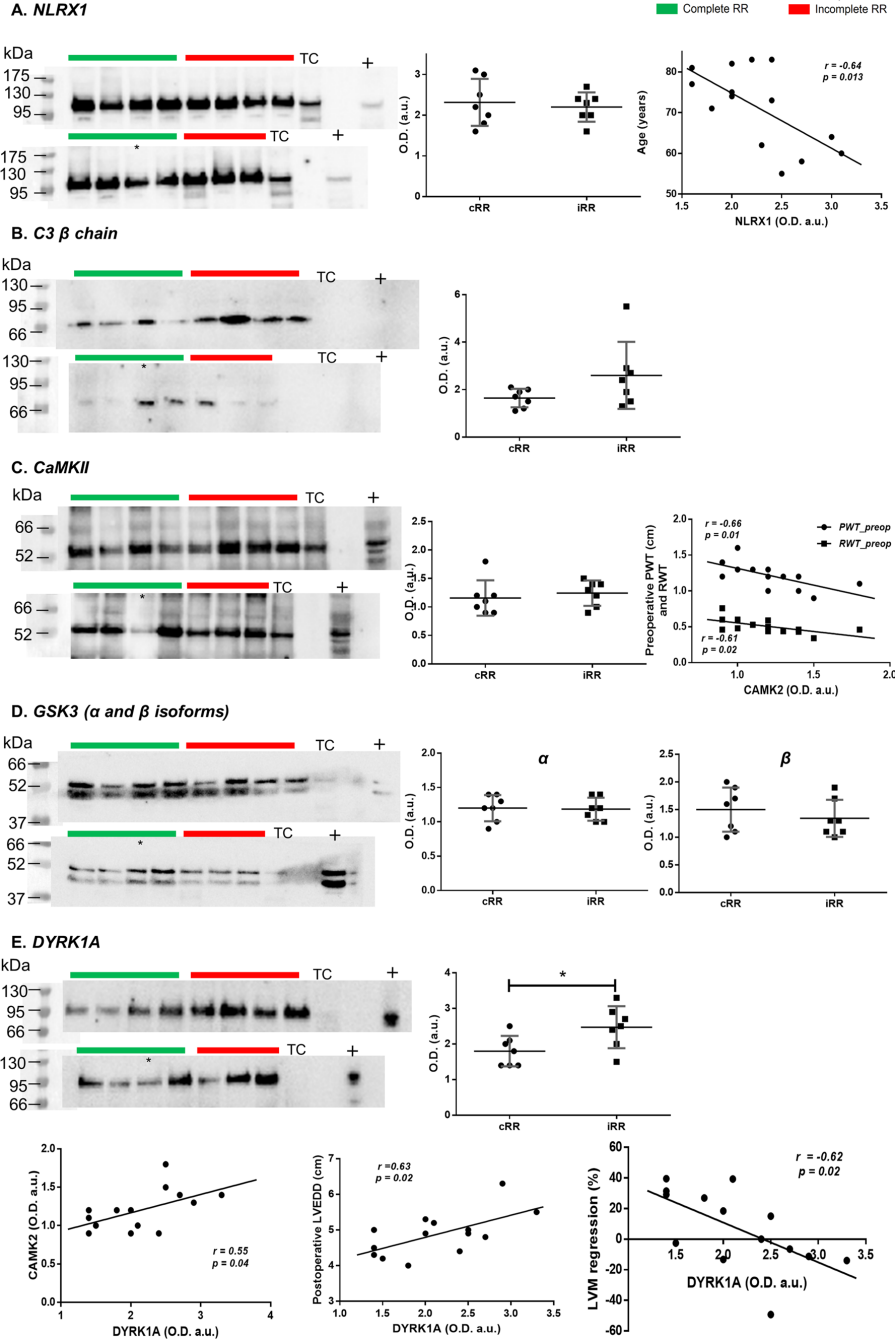
Western blot quantification of (**A**) NLRX1, (**B**) C3 β chain, (**C**) CAMK2, (**D**) GSK3α and β, and (**E**) DYRK1A in patients with complete (*n* = 7) or incomplete (*n* = 7) RR. TC designates a technical control and “ + ” a positive control (NLRX1, GSK3, CAMK2: HeLa cell lysate; C3, DYRK1A: HepG2 lysate for). “*” identifies an excluded sample (aortic insufficiency was found more severe than aortic stenosis). In each blot, the molecular weight of the protein ladder is shown on the left and the respective optical density quantification on the right. Differences were tested by unpaired *t*-test or Mann–Whitney test. Significant correlations between protein levels and clinical variables are also shown. This includes an inverse relationship between age and NLRX1 (**A**, right); an inverse association between CAMK2 and posterior (PWT) and relative wall thickness (RWT) (**C**, right); a direct association between DYRK1A and CAMK2 (**E**, bottom-left), a positive correlation between DYRK1A and postoperative LV end-diastolic dimension (LVEDD) (**E**, bottom-centre) and an inverse correlation between DYRK1A and LVM regression (**E**, bottom-right). In all cases, Pearson’s r is shown, as all variables shown were normal

Considering DEP analysis, kinase prediction and GOEA, some proteins were selected for western blot validation (Fig. 7) in a larger cohort. Correlation with clinical variables was also tested.

Given the reported protective effect in the setting of ischaemia/reperfusion [33], NLRX1 was selected for validation. Notwithstanding the suggested downregulation of NLRX1 expression in iRR patients by proteomics, the assessment in the larger cohort did not support this finding. Nevertheless, an age-associated decrease of NLRX1 was observed (*r* = − 0.64, Fig. 7A). GOEA suggested complement system activation, and complement C3 was one of the components found to increase in the myocardium of iRR patients through proteomics. Therefore, we sought to validate this by immunoblot. Although the intact complement C3 (α + β chain, ~ 185 kDa) could not be immunodetected, probably due to rapid degradation in the lysis buffer, we found a trend for an increase of the C3β chain (remains intact in the activated forms of C3: C3b, iC3b and C3c [34]) in iRR patients (*p* = 0.12, Fig. 7B).

Next, we sought to study the expression of some of the most important predicted kinases. Casein kinase 2 (CK2) and the atypical kinase TAF1 were predicted to be the most active kinases in iRR. Yet, because CK2 is a very broad constitutively active kinase, we instead selected the TAF1 for validation. Nevertheless, TAF1 could not be detected in the human myocardial samples (data not shown), possibly due to low expression. We also selected the pro-hypertrophic kinase calcium/calmodulin-dependent protein kinase II (CAMK2) for validation because it was a common target of the kinases associated with iRR (CK2, TAF1 and IKK, Fig. 6), being phosphorylated in Ser330 and Ser333. Even though no differences in the expression were found between cRR and iRR, high levels of CAMK2 were associated with lower preoperative PWT (*r* = − 0.66) and RWT (*r* = − 0.61, Fig. 7C).

Two kinases with the highest association with cRR, GSK3 and DYRK1A, could successfully be analysed through immunoblot. Using an antibody simultaneously targeting α and β isoforms, the protein levels of the two GSK3 kinases could be measured in the validation cohort. Despite the predicted higher GSK3 activity in cRR, the amount of GSK3α and β did not differ between the two groups (Fig. 7D). Finally, the expression of DYRK1A was 1.4-fold higher (*p* < 0.05) in iRR patients, despite higher predicted activity in cRR patients (Fig. 7E). Of note, a positive association between CAMK2 and DYRK1A was found (*r* = 0.55, Fig. 7E, bottom-left). Remarkably, the preoperative myocardial expression of DYRK1A was associated with a higher propensity for iRR, as demonstrated by the positive correlation with postoperative LVMi (*r* = 0.63, Fig. 7E, bottom-centre) and the negative correlation with LVM regression (*r* = − 0.62, Fig. 7E, bottom-right).

### Uncovering a functional role for DYRK1A

**Fig. 8:**
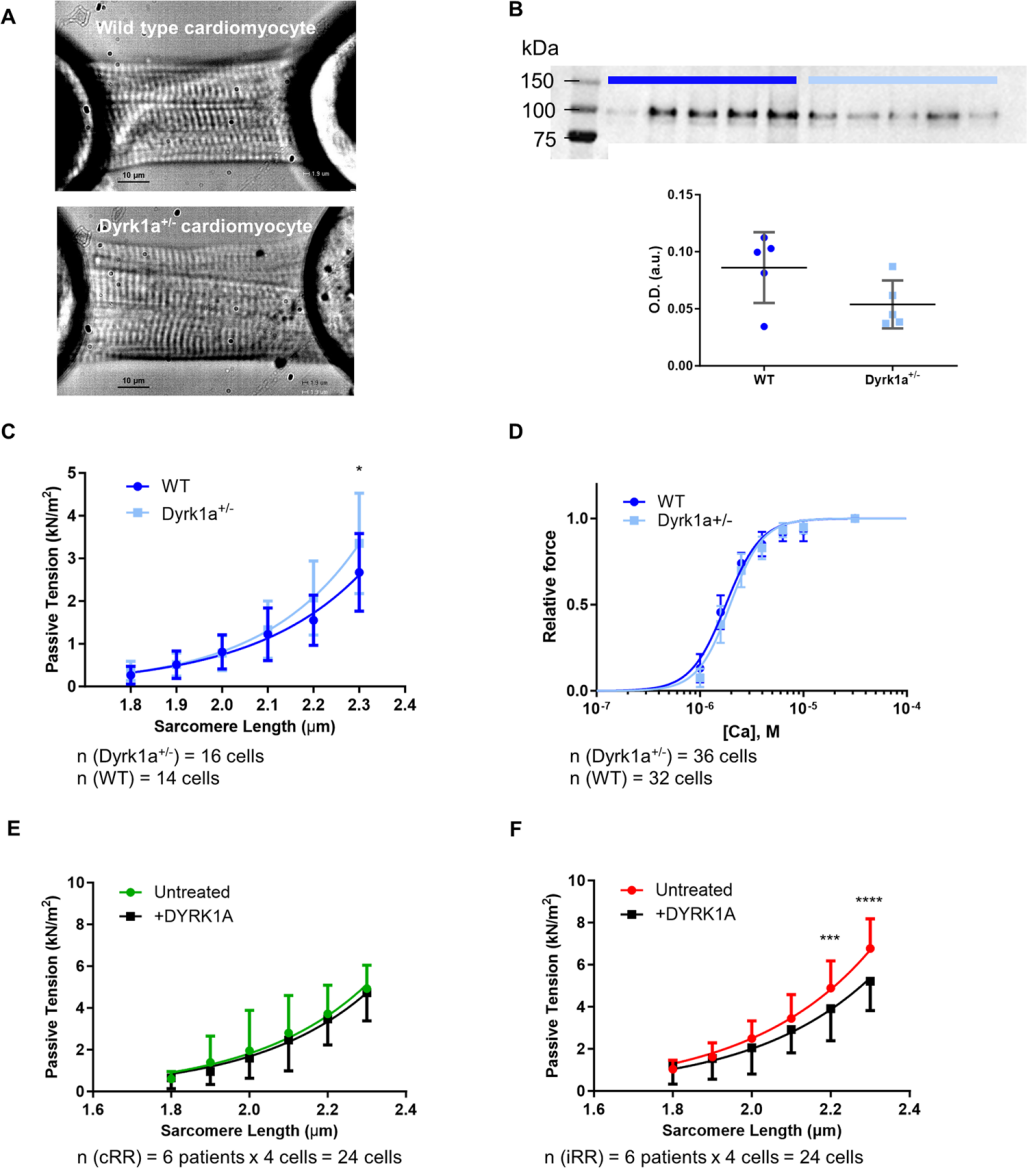
DYRK1A and cardiomyocyte mechanical properties. **A** Snapshot of skinned cardiomyocyte obtained from wild-type and *Dyrk1a*^+/-^ mice. Scale bar, 10 µm. Skinned cardiomyocytes were glued between a force transducer and a motor for the force experiments. **B** Western blot confirmation of Dyrk1a reduced expression in mutant mice (n = 10, *p* < 0.09, unpaired t-test). *Dyrk1a*^+*/*+^ (WT, *n* = 5) and *Dyrk1a*^+/-^ (*n* = 5) mice-derived cardiomyocytes are represented by dark and light blue dots and curves, respectively. **C** SL-PT relationship and **D** calcium sensitivity on isometric (2.2 µm) force development in WT and *Dyrk1a*^+/-^ cardiomyocytes. Force was normalised to that developed at saturating calcium concentration (pCa 4.5). (**E**, **F**) SL–PT relationship in cardiomyocytes from patients with complete (*n* = 6) and incomplete RR (*n* = 6) before (untreated) and after incubation with recombinant DYRK1A (+ DYRK1A). DYRK1A normalised passive tension in incomplete RR to the levels of cardiomyocytes from complete RR. In **E** and **F**, only top or bottom error bars are displayed to improve readability. The differences were inspected with two-way ANOVA with repeated measures, followed by Bonferroni’s multiple comparisons tests

A higher expression of DYRK1A in iRR patients was found by immunoblot quantification despite lower predicted activity (i.e. a lower percentage of phosphorylated events associated). The concomitant dysregulation at the expression and activity levels anticipates a key role during RR, meriting further scrutiny. DYRK1A is constitutively active, pleiotropic and, when ectopically overexpressed, accumulates in the nucleus [35, 36]. The increased expression of DYRK1A in iRR might be a response to hypertrophy, as this kinase directly phosphorylates the pro-hypertrophic nuclear factor of activated T cells (NFAT), favouring its nuclear export [37]. The decreased DYRK1A activity (according to our definition) in iRR may be explained by a potential reduction in the phosphorylation of its cytosolic substrates (i.e. lower pleiotropy). Indeed, DYRK1A interacts with several cytoskeleton proteins, including myofibrillar and Z disc proteins [36], and we confirmed by immunofluorescence that DYRK1A is mainly expressed in the cytoplasm in myocardial tissue of AVS patients (Fig. S2, top panels). In the context of AVS, DYRK1A effects should be limited to the intracellular environment, as no direct association with fibrosis was found (*r* = 0.22, *p* = 0.57, Fig. S2, bottom panels). Hence, we hypothesised that an altered expression of DYRK1A would impact the cardiomyocyte’s contractile function. To dissect DYRK1A’s potential role in cardiomyocyte functional properties, we used permeabilised cardiomyocytes isolated from *Dyrk1a*^+/-^ mice [31] and compared with wild-type *Dyrk1a*^+/+^ littermates (Fig. 8A). The lower expression of DYRK1A in Dyrk1a^+/-^ hearts was confirmed by western blot (Fig. 8B). Two protocols were followed. First, myofibrillar stiffness was studied through the SL–PT relationship. Second, developed force and myofilamentary calcium sensitivity were examined.

#### Sarcomere length–passive tension relationship

The passive tension of each cardiomyocyte was recorded at increasing sarcomere lengths (ranging from 1.8 to 2.3 µm at 0.1 µm-step increases) to obtain the SL–PT relationship (Fig. 8C). In any case, a length-dependent increase in passive tension was observed. Nevertheless, a higher passive tension was observed in *Dyrk1a*^+/-^ animals at higher sarcomere lengths.

#### Development of force and calcium sensitivity

The myofilamentary calcium sensitivity, as quantified by the pCa50 (-log of the half-maximal activating calcium molar concentration), was also studied by recording the force developed at increasing calcium concentrations on a fixed sarcomere length (2.2 µm). There was no difference in calcium sensitivity (pCa_50_: 5.74 ± 0.01 vs. 5.73 ± 0.01, *p* = 0.5, Fig. 8D) or cooperativity (nHill: 2.71 ± 0.01 vs. 2.85 ± 0.08, *p* = 0.3, Table 4) between wild-type and mutant mice. Nonetheless, *Dyrk1a*^+/-^-derived myocytes showed a significant decrease in the developed (active) tension under calcium-saturating conditions (pCa 4.5) (Table 4).

**Table 4:** Mechanical properties of the skinned cardiomyocytes retrieved from the force development (calcium sensitivity) protocol

Mechanical properties	WT (*n* = 32, 5 animals)	DYRK1A^+/-^(*n* = 36, 5 animals)	*p*-value
Total tension (kN.m^−2^)	24.2 ± 12.1	18.1 ± 11.5	**
Passive tension (kN.m^−2^)	1.2 ± 1.0	1.0 ± 1.3	
Active tension (kN.m^−2^)	23.0 ± 11.8	17.1 ± 10.4	*
Maximal active tension (kN.m^−2^)	38.9 ± 8.4	31.6 ± 7.9	
k_tr_ (s^−1^)	15.3 ± 2.4	14.4 ± 4.0	
Residual force	0.67 ± 0.10	0.86 ± 0.82	#
pCa_50_	5.74 ± 0.01	5.73 ± 0.01	
nHill	2.71 ± 0.11	2.85 ± 0.08	

Data is presented as mean ± SD. * *p* < 0.05 ** *p* < 0.01 (data was log-transformed; unpaired two-tailed *t*-test); # *p* < 0.05 (Mann–Whitney test)

*k*_*tr*_ rate of force redevelopment, *nHill* steepness of the force–pCa relationship, *pCa*_*50*_ -log([Ca^2+^]), for which developed force is half of maximal

#### Effect of DYRK1A treatment on patients-derived cardiomyocytes

Provided the effect of DYRK1A on myofibrillar stiffness in the *Dyrk1a*^+/-^ mouse model, and considering that stiffness is a significant contributor to diastolic dysfunction, we aimed to test the effect of DYRK1A treatment in cardiomyocytes isolated from an independent population of AVS patients (Table 1). Thus, cardiomyocytes from six cRR and six iRR patients were isolated and incubated with recombinant DYRK1A or relaxing solution before obtaining the SL–PT relationship (Fig. 8E, F). While no effect was observed on patients with cRR, adding DYRK1A to the cardiomyocytes of iRR patients reduced passive tension to the levels of cRR patients.

## Discussion

Despite the immediate elimination of the pressure afterload upon valve replacement, in many patients, myocardial hypertrophy and diastolic dysfunction are sustained in time, increasing the risk of HF and death [5, 38]. A complete comprehension of the molecular players and pathways in the inception of an iRR is imperative. First, it will help define the best timing for intervention, aiming for the most complete RR. Second, it will open new treatment avenues to accelerate recovery. Previously, proteomics helped identify many factors involved in myocardial (reverse) remodelling in animal models of aortic banding, including fibrosis, metabolic derangement and mitochondrial antioxidant defences [11, 12, 17]. Herein, we characterised, for the first time, the myocardial proteome in AVS patients with divergent RR phenotypes. We further explored the phosphoproteome to uncover kinases as new therapeutic targets in iRR and highlighted DYRK1A as a potential target in iRR.

One of the most striking differences between cRR and iRR patients resided in the activation of the immune system, particularly the humoral arm, in addition to the complement system. Complement factors such as C3 and C4 might be implicated in the clearance of apoptotic cardiomyocytes, according to a finer GOEA (GO term “positive regulation of apoptotic cell clearance”, Fig. 2A). We found a trend for higher myocardial expression of the C3 β chain in iRR, suggesting the involvement of complement in regulating cardiomyocyte death under pressure overload. In fact, higher levels of complement C9 and the associated oncosis (ischaemic cell death) were described in patients undergoing AVR [39]. Previously, targeting the complement system (complement C5a receptor) was shown to prevent cardiac remodelling (hypertrophy, inflammation, and fibrosis) in a hypertension model [40].

Given the postmitotic state of cardiomyocytes, AVR-driven hypertrophy reversal relies on the ubiquitin–proteasome system (UPS) and autophagy [41]. Our proteomics data shows an increased expression of UPS players in cRR patients, such as the E3 ubiquitin ligase HUWE1 or the proteasome proteins PSME2 and PSMD1. This suggests a higher rate of protein recycling in cRR patients and is supported by previously reported correlations between E3 ubiquitin ligases, such as atrogin-1 and MuRF1, and postoperative hypertrophy [42]. HUWE1 was also found to be reduced in cardiomyopathy patients with end-stage HF [43].

Myocardial proteomics also evidenced metabolic differences between cRR and iRR patients, mainly affecting mitochondria. Most upregulated proteins in cRR are in the mitochondrial matrix, part of oxidoreductase complexes or involved in acetyl-CoA synthesis. A relatively lower content of mitochondrial proteins in iRR suggests worse myocardial bioenergetics. Therefore, we modelled myocardial metabolism [27] with an increasing energetic demand to simulate the increased workload-driven energetic expenditure which comes with stenosis aggravation. We confirmed that, under high energetic demand, fatty acids remain the main energetic substrate in both conditions, but the preference is always higher in patients with cRR. Patients with iRR, in turn, were predicted to rely less on aerobic metabolism and, therefore, consume less oxygen and generate less ATP. This metabolic handicap may explain their worse outcome (hypertrophy). These observations require validation in vivo*,* but according to our aortic banding model, this should entail unbalanced mitochondria dynamics, where fission is favoured over biogenesis [44].

A myriad of signalling pathways governs myocardial RR. We used the phosphoproteome data to predict the leading players in such pathways and uncover new therapeutic targets for iRR. CK2, TAF1 and the IKK family were the kinases most associated with iRR. CK2 is a pleiotropic kinase that regulates cell cycle progression, apoptosis and transcription (UniProt). The association with iRR strengthens previous evidence of an aortic banding murine model, where a highly phosphorylated motif (pSDxD) easily recognised by CK2 (pS/TxxD/E) was found in hypertrophied hearts [13]. Thus, CK2 inhibition may hold therapeutic value in iRR. In turn, TAF1 is a TATA-box-binding protein which forms the core scaffold of the basal transcription initiation factor IID (TFIID). TAF1’s predicted higher activity in iRR might be a response to the hypertrophic stimulus. Indeed, hypertrophic agonists like phenylephrine induce TAF1 expression in neonatal rat cardiomyocytes [45]. Finally, the IKK family comprises a group of kinases that prevent nuclear factor kappa B (NF-κB) degradation and, thus, favour pro-inflammatory and pro-hypertrophic gene expression. NF-κB role in pathological hypertrophy is well established in pressure overload mouse models [15]. Interestingly, CAMK2 was found to be a target of the CK2, TAF1 and the IKK family. Although no difference in CAMK2 expression was observed between RR phenotypes, CAMK2 was inversely correlated with PWT and RWT, corroborating the established association between CAMK2 higher expression and LV dilation [46].

On the opposite side, we found GSK3, DYRK2 and DYRK1A most associated with cRR. GSK3α and β share a highly identical kinase domain and may both reduce hypertrophy in pressure overload [47, 48]. In turn, DYRK2 and DYRK1A belong to a family of highly conserved kinases, also known as proline-directed kinases [35]. There is in vitro evidence of a hypertrophy-limiting role of DYRK2 and DYRK1A. For instance, DYRK1A inhibited cardiomyocyte hypertrophy in response to phenylephrine and calcineurin overexpression [37]. Herein, we found higher DYRK1A protein levels in patients evolving to an iRR phenotype. Yet, at the time of DYRK1A assessment (during AVR), iRR patients presented lower LVMi (130.9 ± 23.2 vs 162.5 ± 36.9 g/m^2^ in cRR, *p* = 0.08). Hence, while the association of higher DYRK1A myocardial levels with lower hypertrophy in AVS was confirmed, we were intrigued by the inverse correlation with LVM regression. The most probable explanation is that while DYRK1A upregulation is part of an intrinsic myocardial mechanism of negative feedback to NFAT overactivation that aims to prevent excess hypertrophy during pressure overload [35, 37], in the long-term, sustained DYRK1A upregulation may fail to inhibit NFAT in some patients, who end up showing worse outcomes, remarkably persistent hypertrophy (iRR). This is strongly corroborated by Grebe et al*.* [49], who showed that while DYRK1A successfully inhibited acute NFAT activation in cardiomyocytes in vitro, it failed to prevent hypertrophy and led to the activation of maladaptive genes in a mice model of aortic banding-induced (chronic) hypertrophy. In another study, Dyrk1a overexpression was detrimental to mice heart development, impairing cardiomyocyte proliferation and resulting in dilated cardiomyopathy [50]. In humans, increased dosage of DYRK1A, such as in Down syndrome, leads to congenital heart defects and reduced expression of mitochondrial respiration genes [51]. Since our study does not establish causality, a future challenge will be identifying the factors associated with the loss of DYRK1A’s protective effect in some patients. According to the analysis of the myocardial proteome, proteostasis dysregulation and metabolic underperformance arise as priority candidates.

Following the observation of an increased myocardial DYRK1A expression, but paradoxically reduced activity (i.e. with lower diversity of targets) in patients evolving to iRR, and given the interaction of DYRK1A with sarcomeric proteins [36], we hypothesised that the dysregulation of DYRK1A in RR had functional consequences, particularly, in cardiomyocyte contractility. Therefore, we first compared contractile properties between wild-type *Dyrk1a*^+/+^ and *Dyrk1a*^+/-^ mice cardiomyocytes. Our data extends the previously known pro-hypertrophic effect of Dyrk1a haploinsufficiency in mice under pressure overload [52] and suggests that these mice have higher stiffness as assessed by the significant interaction between genotype and the SL–PT curve, higher passive tension when cardiomyocytes were stretched to 2.2 µm and higher residual force when exposed to increasing calcium concentrations. The latter may reflect a greater difficulty in actomyosin detachment, i.e. impaired relaxation [53]. As for active tension, DYRK1A expression does not significantly reduce maximal active force or modify calcium sensitivity, in line with previous studies showing that calcium sensitivity does not seem to be affected in AVS [54].

The present model of DYRK1A’s reduced expression has a parallel in the pre-AVR pro-hypertrophic state, where DYRK1A is diverted to the mitigation of NFAT-mediated expression of hypertrophic genes [37], limiting its cytosolic pleiotropy. For this reason, we tested the effect of DYRK1A incubation on AVS patient-derived cardiomyocytes. DYRK1A did not change the SL–PT relationship in cRR patients, which suggests the conservation of DYRK1A activity. Interestingly, cardiomyocytes from iRR patients presented higher passive tensions, especially pronounced at higher sarcomere lengths, which were rescued upon in vitro incubation with DYRK1A. This suggests that despite the higher expression, DYRK1A activity at the sarcomeric level is depressed in patients with iRR, resulting in higher myofibrillar stiffness (adding to the long-term failure to mitigate NFAT-mediated hypertrophy). From the phosphoproteome data, DYRK1A was predicted to target troponin I on Ser183 (decreased in iRR patients). Troponin I interacts with all known regulatory proteins in the thin filament (troponins T and C, tropomyosin and actin) [55]. Therefore, a higher passive tension in iRR cardiomyocytes may result from a deficient troponin complex stabilisation required to prevent actomyosin complex assembly at diastolic calcium concentrations [56]. Altogether, this suggests that DYRK1A dysregulation may contribute to and can be a target to treat hypertrophy and diastolic dysfunction in AVS patients.

This study has the limitations of a retrospective approach, preventing us from inferring causality from the association between DYRK1A expression and RR. To avoid confounding effects on myocardial remodelling, patients with severe aortic regurgitation, other severe valve diseases or previous infarction were excluded. A group of patients were selected that could minimise comorbidities-related bias. We cannot fully exclude the influence of obesity in the discovery cohort (A), but for the validation cohorts (B and C), all important comorbidities were balanced. Although not statistically significant, most iRR patients in cohorts A and B were women. The influence of the patient’s sex in myocardial phosphoproteome will require further scrutiny, but the final DYRK1A assays were conducted with a sex-balanced group of iRR patients. Heterogeneity in the timing elapsed between surgery and postoperative echocardiography may influence the evaluation of the RR. Notwithstanding, the bulk of LVM regression, this study’s primary outcome, was completed within 6 months post-AVR [3], and our cohorts presented a median difference of 9 and 7 months. The bioinformatic approach to phosphoproteome data is cell blind, precluding an assignment of dysregulated kinases to specific cell types. Signalling pathways are also regulated by phosphatases, whose prediction was outside the scope of this work. In addition, we used permeabilised cells, which are a membrane- and organelles-free simplified model of cardiomyocytes. Thus, the effects of DYRK1A upregulation and downregulation must be confirmed in intact cells and in vivo.

## Conclusion

This study is the first to characterise the myocardial (phospho)proteome in patients showing divergent myocardial responses to AVR. Better myocardial bioenergetics, with conservation of fatty acid oxidation by mitochondria, may underscore a complete regression of LVM. Instead, patients with myocardial signs of complement, inflammatory and immune pathways activation may be primed for an incomplete RR. Phosphoproteome-based kinase prediction identified DYRK1A as one of the main dysregulated kinases between patients with complete and incomplete RR. Perioperative DYRK1A myocardial levels are inversely correlated with LVM regression. Despite its known anti-hypertrophic role, our data suggests that chronic elevation of DYRK1A may have detrimental consequences in AVS patients. Patients who develop incomplete RR show increased basal myocardial stiffness, which normalises upon DYRK1A supplementation. DYKR1A emerges as a surrogate target to treat AVS patients showing postoperative diastolic dysfunction.

## Conflicts of interest

The authors declare that they have no conflicts of interest.

## Ethical approval

This study was approved by the Ethics Committee of Centro Hospitalar Universitário de São João (Ref 109/2022) and abided by the 1964 Declaration of Helsinki and its later amendments. Informed consent was obtained from all patients.

## Supplementary Information

Below is the link to the electronic supplementary material.

Supplementary file 1 (PDF 898 KB)

## Data Availability

The proteome and phosphoproteome data have been deposited at the ProteomeXchange Consortium via PRIDE partner repository, respectively, with the dataset identifiers PXD015497 and PXD015498. The remaining data are available in the article and in its online supplementary material.
