# Supplementary material for: Myocardial phosphoproteomics unveils a key role of DYRK1A in aortic valve replacement-induced reverse remodelling
Source: Basic Res Cardiol. 2025 Jul 6;120(5):947–74. doi: 10.1007/s00395-025-01125-w (PMC12518468; doi:10.1007/s00395-025-01125-w)
Supplement: Supplementary file 1 — (PDF 898 KB) [file 395_2025_1125_MOESM1_ESM.docx]

**Basic Research in Cardiology**

Myocardial phosphoproteomics unveils a key role of DYRK1A in aortic valve replacement-induced reverse remodelling

Fábio Trindade^1^, João Almeida-Coelho^1^, Cláudia Sousa-Mendes^1^, Francisca Saraiva^1^, Maria L. Arbonés^2,3^, Adelino Leite-Moreira^1^, Rui Vitorino^1,4,5 *^, Inês Falcão-Pires^1 *^

* equal contribution - senior authors

^1^ Cardiovascular R&D Centre—UnIC@RISE, Department of Surgery and Physiology, Faculty of Medicine, University of Porto, 4200-319 Porto, Portugal

^2^ IBMB-CSIC – Institut de Biologia Molecular de Barcelona, 08028 Barcelona, Spain

^3^ CIBERER – Centro de Investigación Biomédica en Red de Enfermedades Raras, 08028 Barcelona, Spain

^4^ iBiMED – Institute of Biomedicine, Department of Medical Sciences, University of Aveiro, 3810-193 Aveiro, Portugal

^5^ LAQV/REQUIMTE, Department of Chemistry, University of Aveiro, 3810-193 Aveiro, Portugal

Corresponding author:

Inês Falcão-Pires ([ipires@med.up.pt](mailto:ipires@med.up.pt))

Phone: +351 220 426 805

Cardiovascular R&D Centre - UnIC@RISE

Department of Surgery and Physiology, Faculty of Medicine of the University of Porto

4200-319 Porto, Portugal

**Supplementary Figures**

**Fig. S1** Overview of the left ventricle mass (LVM) regression (%) in the population initially screened for this study (n = 77). Data density analysis showed that the median LVM regression was around 15% of the preoperative LVM. We set 15% as the cut-off for a complete myocardial reverse remodelling. Less than 25% of the patients presented a regression below 5%, and this condition was defined as an incomplete myocardial reverse remodelling. A 10% gap was allowed to emphasise phenotypical differences and facilitate the discovery of molecular targets.


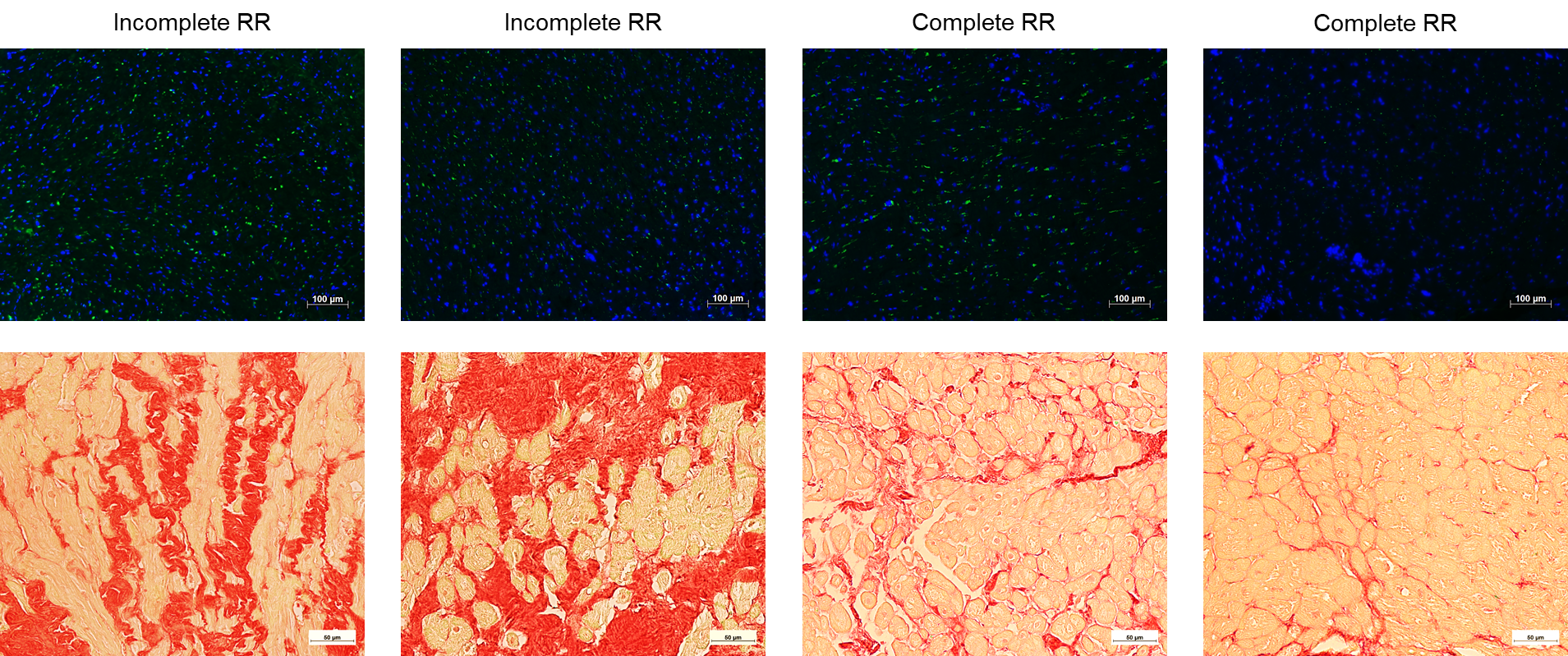


**Fig. S2** Myocardial expression of DYRK1A (top) and myocardial fibrosis (bottom). 4 representative images are displayed for each stain. DYRK1A was detected by immunofluorescence in 9 myocardial tissue sections from AVS patients with complete (n=4) or incomplete (n=5) reverse remodelling. Nuclei are stained in blue (DAPI) and DYRK1A is stained in green (FITC). DYRK1A shows a low degree of colocalization with DAPI, suggesting that it is mainly found in the cytoplasm. Fibrosis was evaluated in the same sections by Picro Sirius Red staining. No correlation was found between DYRK1A expression and the extent of fibrosis (r=0.22, p=0.57).


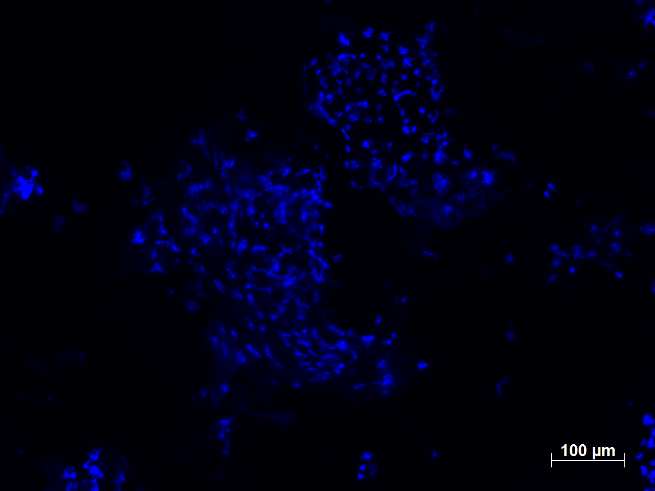

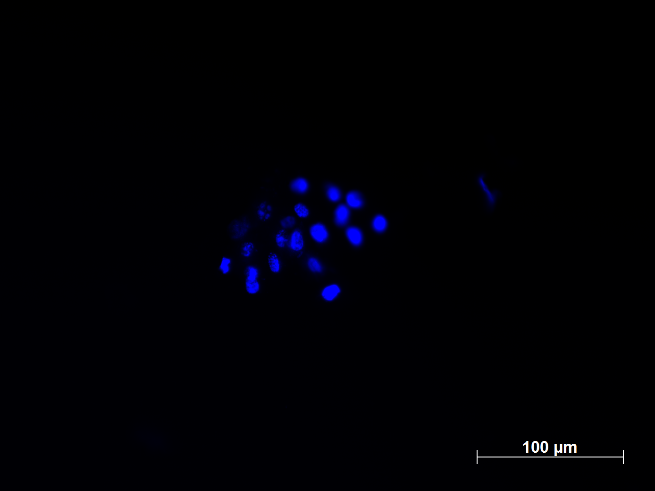


**Fig. S3** The effect of Triton X-100 on nuclei integrity. When cardiac tissue homogenates are incubated with 0.1% Triton X-100 for 5 minutes on ice, nuclei lose their integrity, as shown by the diffuse DAPI staining (right). Nuclei preserve a perfect round shape when incubated with isolation buffer without this detergent (left). This treatment is essential to obtain skinned cardiomyocytes. In this model, most steps required for force generation *in vivo* are eliminated, while force can still be generated (when all cofactors required for contraction are provided) and recorded in microscopes equipped with an electromagnetic motor and a force transducer. This robust system is often used to test drug effects on cardiomyocyte contractility at the early phases of drug development.


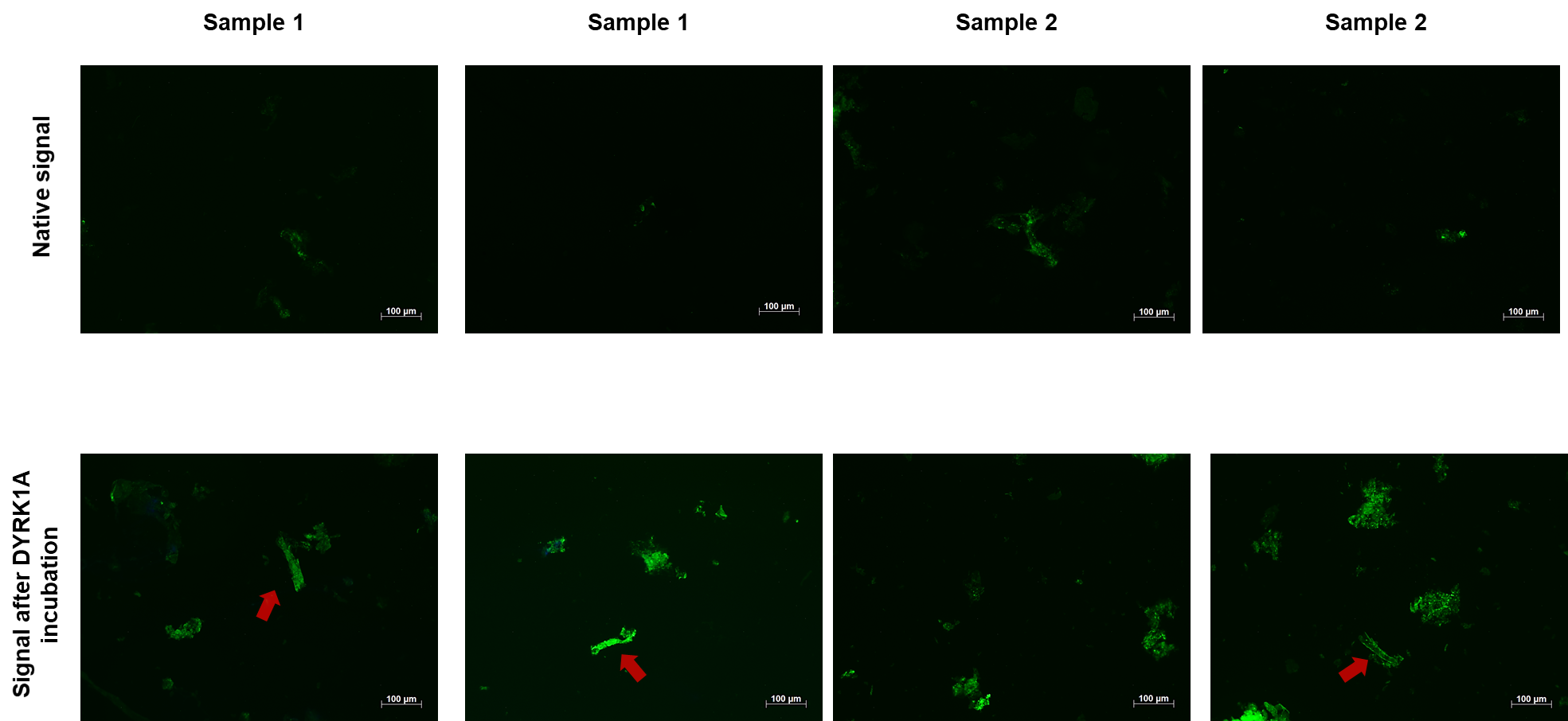


**Fig. S4** Demonstration of recombinant DYRK1A internalisation in permeabilised cardiomyocytes. Cardiomyocytes were isolated from two different samples and permeabilised using the same treatment for force recording experiments. A fraction of the cell suspensions was incubated with recombinant DYRK1A (bottom panel) or with the same volume of the relaxing solution to measure the native signal (control, top panel). Then, cells were fixed for 15 minutes with 4% paraformaldehyde, washed 3 times with PBS, incubated 60 minutes with a fluorescent anti-DYRK1A antibody (fluorescein isothiocyanate) at 4°C in the dark, washed 3 times with PBS and analysed with a fluorescence microscope. A stronger DYRK1A signal was evident in the fraction incubated with the recombinant protein, demonstrating its internalisation. Red arrows point to cardiomyocytes that would be candidates for force recording. In these cells, DYRK1A signal was scattered throughout the cell.

**Supplementary Tables**

**Table S1.** Immunoblot specific conditions.

| **Protein target** | **UniProt ID and Gene Name** | **Epitope^a^** | **Application** | **Blocking conditions** | **Primary antibody** | **Incubation conditions** | **Secondary antibody** | **Incubation conditions** |
| --- | --- | --- | --- | --- | --- | --- | --- | --- |
| NLR family member X1 | Q86UT6  NLRX1 or NOD9 | 89-230 (human) | WB | 1 hour in 5% non-fat milk in TBS | Mouse mAb 1:1000; sc-7294 | Overnight  4 °C | HRP-linked sheep anti-mouse 1:5000; GE Amersham NA931 | 1 hour  RT |
| Complement C3 | P01024  C3 | 541-840 (human) | WB | 1 hour in 5% non-fat milk in TBS-T | Mouse mAb 1:1000; sc-28294 | Overnight  4 °C | HRP-linked sheep anti-mouse 1:5000; GE Amersham NA931 | 1 hour  RT |
| Calcium/calmodulin-dependent protein kinase type II^b^ | Not specific to a CAMK2 isoform | Full length protein (rat) | WB | 2 hours in 5% non-fat milk in TBS-T | Mouse mAb 1:1000; ab22609 | Overnight  4 °C | HRP-linked sheep anti-mouse 1:5000; GE Amersham NA931 | 1 hour  RT |
| Glycogen synthase kinase-3 α and β^b^ | P49840/ P49841  GSK3A/GSKB | Full length protein (*Xenopus laevis*) | WB | Overnight in 5% non-fat milk in TBS-T | Mouse mAb 1:500; Invitrogen #44-610 | 2 hours  RT | HRP-linked sheep anti-mouse 1:5000; GE Amersham NA931 | 1 hour  RT |
| Dual specificity tyrosine-phosphorylation-regulated kinase 1A | Q13627  DYRK1A | Full length protein (human) | WB | 1 hour in 5% non-fat milk in TBS-T | Mouse mAb 1:1000; sc-100376 | Overnight  4 °C | HRP-linked sheep anti-mouse 1:5000; GE Amersham NA931 | 1 hour  RT |

^a^ Target residues range, according to the datasheet

Abbreviations: HRP: horseradish peroxidase; mAb: monoclonal antibody; RT: room temperature; TBS: Tris(hydroxymethyl)aminomethane buffer saline; TBS-T: TBS-Tween-20; WB: western blot

**Table S2.** List of enriched gene ontology terms related to biological processes, molecular functions and cellular components. The terms are sorted in descending order of gene ratio, followed by adjusted p-value.

| **GO Term** | **Description** | **Gene Ratio** | **Background Ratio** | **Adjusted p-value** | **Gene IDs** |
| --- | --- | --- | --- | --- | --- |
| *Biological Processes - Complete Reverse Remodelling* | | | | | |
| GO:0018335 | protein succinylation | 02/55 | 4/18722 | 0.026 | DLD; OGDH |
| GO:0106077 | histone succinylation | 02/55 | 4/18722 | 0.026 | DLD; OGDH |
| GO:0061732 | mitochondrial acetyl-CoA biosynthetic process from pyruvate | 02/55 | 5/18722 | 0.029 | PDHX; DLD |
| *Biological Processes - Incomplete Reverse Remodelling* | | | | | |
| GO:0002253 | activation of immune response | 16/89 | 375/18722 | <0.001 | IGHV3-49; CRP; C1QC; CD5L; C1QB; C1S; PRKDC; IFI16; C4B; C4A; IGHG4; IGHM; CFHR1; C4BPA; C3; SFPQ |
| GO:0006959 | humoral immune response | 15/89 | 317/18722 | <0.001 | IGHV3-49; CRP; C1QC; CD5L; IGKV3-20; C1QB; C1S; C4B; C4A; IGHG4; IGHM; CFHR1; C4BPA; C3; HRG |
| GO:0006956 | complement activation | 13/89 | 130/18722 | <0.001 | IGHV3-49; CRP; C1QC; CD5L; C1QB; C1S; C4B; C4A; IGHG4; IGHM; CFHR1; C4BPA; C3 |
| GO:0006958 | complement activation, classical pathway | 11/89 | 108/18722 | <0.001 | IGHV3-49; CRP; C1QC; C1QB; C1S; C4B; C4A; IGHG4; IGHM; C4BPA; C3 |
| GO:0002455 | humoral immune response mediated by circulating immunoglobulin | 11/89 | 121/18722 | <0.001 | IGHV3-49; CRP; C1QC; C1QB; C1S; C4B; C4A; IGHG4; IGHM; C4BPA; C3 |
| GO:0016064 | immunoglobulin mediated immune response | 11/89 | 207/18722 | <0.001 | IGHV3-49; CRP; C1QC; C1QB; C1S; C4B; C4A; IGHG4; IGHM; C4BPA; C3 |
| GO:0019724 | B cell mediated immunity | 11/89 | 210/18722 | <0.001 | IGHV3-49; CRP; C1QC; C1QB; C1S; C4B; C4A; IGHG4; IGHM; C4BPA; C3 |
| GO:0002449 | lymphocyte mediated immunity | 11/89 | 350/18722 | <0.001 | IGHV3-49; CRP; C1QC; C1QB; C1S; C4B; C4A; IGHG4; IGHM; C4BPA; C3 |
| GO:0045861 | negative regulation of proteolysis | 11/89 | 351/18722 | <0.001 | LRP1; IFI16; C4B; C4A; SERPINF2; EIF3H; ITIH4; C3; HRG; AGT; TMED10 |
| GO:0002460 | adaptive immune response based on somatic recombination of immune receptors built from immunoglobulin superfamily domains | 11/89 | 356/18722 | <0.001 | IGHV3-49; CRP; C1QC; C1QB; C1S; C4B; C4A; IGHG4; IGHM; C4BPA; C3 |
| GO:0002443 | leukocyte mediated immunity | 11/89 | 440/18722 | <0.001 | IGHV3-49; CRP; C1QC; C1QB; C1S; C4B; C4A; IGHG4; IGHM; C4BPA; C3 |
| GO:0022411 | cellular component disassembly | 11/89 | 443/18722 | <0.001 | C1QC; GSPT1; C1QB; MMP2; CAPG; LRP1; GFM2; TMOD3; C3; SPTB; SET |
| GO:0010466 | negative regulation of peptidase activity | 10/89 | 262/18722 | <0.001 | LRP1; IFI16; C4B; C4A; SERPINF2; ITIH4; C3; HRG; AGT; TMED10 |
| GO:0006909 | phagocytosis | 10/89 | 308/18722 | <0.001 | IGHV3-49; CRP; CD14; LRP1; C4B; C4A; IGHG4; IGHM; C4BPA; C3 |
| GO:0051346 | negative regulation of hydrolase activity | 10/89 | 379/18722 | <0.001 | LRP1; IFI16; C4B; C4A; SERPINF2; ITIH4; C3; HRG; AGT; TMED10 |
| GO:0052547 | regulation of peptidase activity | 10/89 | 461/18722 | 0.010 | LRP1; IFI16; C4B; C4A; SERPINF2; ITIH4; C3; HRG; AGT; TMED10 |
| GO:0010951 | negative regulation of endopeptidase activity | 9/89 | 252/18722 | <0.001 | IFI16; C4B; C4A; SERPINF2; ITIH4; C3; HRG; AGT; TMED10 |
| GO:0032103 | positive regulation of response to external stimulus | 9/89 | 427/18722 | 0.010 | PRKDC; LRP1; IFI16; USP15; SERPINF2; C3; HRG; AGT; SFPQ |
| GO:0052548 | regulation of endopeptidase activity | 9/89 | 432/18722 | 0.010 | IFI16; C4B; C4A; SERPINF2; ITIH4; C3; HRG; AGT; TMED10 |
| GO:0042742 | defense response to bacterium | 7/89 | 350/18722 | 0.050 | IGHV3-49; SSC5D; CRP; PGLYRP2; IGKV3-20; IGHG4; IGHM |
| GO:0006910 | phagocytosis, recognition | 6/89 | 100/18722 | <0.001 | IGHV3-49; CRP; C4B; IGHG4; IGHM; C4BPA |
| GO:0002526 | acute inflammatory response | 6/89 | 112/18722 | <0.001 | CRP; KLKB1; CD163; SERPINF2; ITIH4; C3 |
| GO:0007623 | circadian rhythm | 6/89 | 210/18722 | 0.020 | PRKDC; THRAP3; HNRNPR; SFPQ; PRMT5; HNRNPD |
| GO:0032984 | protein-containing complex disassembly | 6/89 | 224/18722 | 0.030 | GSPT1; CAPG; GFM2; TMOD3; SPTB; SET |
| GO:0008037 | cell recognition | 6/89 | 225/18722 | 0.030 | IGHV3-49; CRP; C4B; IGHG4; IGHM; C4BPA |
| GO:0051702 | biological process involved in interaction with symbiont | 5/89 | 94/18722 | 0.010 | CRP; IGF2R; STOM; CFHR1; HRG |
| GO:0043624 | cellular protein complex disassembly | 5/89 | 136/18722 | 0.020 | GSPT1; CAPG; GFM2; TMOD3; SPTB |
| GO:0032200 | telomere organization | 5/89 | 159/18722 | 0.040 | PRKDC; TNKS1BP1; NEK7; H4C1; HNRNPD |
| GO:0002833 | positive regulation of response to biotic stimulus | 5/89 | 168/18722 | 0.050 | PRKDC; IFI16; USP15; HRG; SFPQ |
| GO:0006953 | acute-phase response | 4/89 | 49/18722 | 0.010 | CRP; CD163; SERPINF2; ITIH4 |
| GO:0043277 | apoptotic cell clearance | 4/89 | 52/18722 | 0.010 | LRP1; C4B; C4A; C3 |
| GO:0031638 | zymogen activation | 4/89 | 60/18722 | 0.010 | KLKB1; CD5L; IFI16; SERPINF2 |
| GO:0050766 | positive regulation of phagocytosis | 4/89 | 66/18722 | 0.010 | LRP1; C4B; C4A; C3 |
| GO:0050764 | regulation of phagocytosis | 4/89 | 95/18722 | 0.040 | LRP1; C4B; C4A; C3 |
| GO:0045807 | positive regulation of endocytosis | 4/89 | 100/18722 | 0.050 | CD14; LRP1; C3; AP2A1 |
| GO:2000427 | positive regulation of apoptotic cell clearance | 3/89 | 8/18722 | <0.001 | C4B; C4A; C3 |
| GO:2000425 | regulation of apoptotic cell clearance | 3/89 | 10/18722 | <0.001 | C4B; C4A; C3 |
| GO:0098883 | synapse pruning | 3/89 | 11/18722 | <0.001 | C1QC; C1QB; C3 |
| GO:0008228 | opsonization | 3/89 | 12/18722 | <0.001 | CRP; C4B; C4BPA |
| GO:0051917 | regulation of fibrinolysis | 3/89 | 14/18722 | <0.001 | KLKB1; SERPINF2; HRG |
| GO:0030449 | regulation of complement activation | 3/89 | 21/18722 | 0.010 | CD5L; C4BPA; C3 |
| GO:0150146 | cell junction disassembly | 3/89 | 21/18722 | 0.010 | C1QC; C1QB; C3 |
| GO:0042730 | fibrinolysis | 3/89 | 25/18722 | 0.010 | KLKB1; SERPINF2; HRG |
| GO:0098581 | detection of external biotic stimulus | 3/89 | 25/18722 | 0.010 | SSC5D; PGLYRP2; C4B |
| GO:0044788 | modulation by host of viral process | 3/89 | 30/18722 | 0.020 | CRP; IGF2R; STOM |
| GO:0009595 | detection of biotic stimulus | 3/89 | 38/18722 | 0.030 | SSC5D; PGLYRP2; C4B |
| GO:0016233 | telomere capping | 3/89 | 39/18722 | 0.040 | PRKDC; NEK7; HNRNPD |
| GO:0051693 | actin filament capping | 3/89 | 41/18722 | 0.040 | CAPG; TMOD3; SPTB |
| GO:0002920 | regulation of humoral immune response | 3/89 | 45/18722 | 0.050 | CD5L; C4BPA; C3 |
| GO:0030835 | negative regulation of actin filament depolymerization | 3/89 | 45/18722 | 0.050 | CAPG; TMOD3; SPTB |
| GO:0002034 | maintenance of blood vessel diameter homeostasis by renin-angiotensin | 2/89 | 4/18722 | 0.010 | SERPINF2; AGT |
| GO:0003072 | renal control of peripheral vascular resistance involved in regulation of systemic arterial blood pressure | 2/89 | 4/18722 | 0.010 | SERPINF2; AGT |
| GO:0051715 | cytolysis in other organism | 2/89 | 9/18722 | 0.030 | CFHR1; HRG |
| GO:0051801 | cytolysis in other organism involved in symbiotic interaction | 2/89 | 9/18722 | 0.030 | CFHR1; HRG |
| GO:0051838 | cytolysis by host of symbiont cells | 2/89 | 9/18722 | 0.030 | CFHR1; HRG |
| GO:0051918 | negative regulation of fibrinolysis | 2/89 | 10/18722 | 0.040 | SERPINF2; HRG |
| GO:0097278 | complement-dependent cytotoxicity | 2/89 | 10/18722 | 0.040 | CD5L; C3 |
| GO:0032493 | response to bacterial lipoprotein | 2/89 | 11/18722 | 0.050 | SSC5D; CD14 |
| GO:0032490 | detection of molecule of bacterial origin | 2/89 | 12/18722 | 0.050 | SSC5D; C4B |
| *Molecular Functions – Complete Reverse Remodelling* | | | | | |
| GO:0016620 | oxidoreductase activity, acting on the aldehyde or oxo group of donors, NAD or NADP as acceptor | 4/57 | 37/18367 | <0.001 | ADH5; PDHX; DLD; OGDH |
| GO:0016903 | oxidoreductase activity, acting on the aldehyde or oxo group of donors | 4/57 | 44/18367 | <0.001 | ADH5; PDHX; DLD; OGDH |
| GO:0008135 | translation factor activity, RNA binding | 4/57 | 84/18367 | 0.004 | DENR; EIF1B; EEF1A2; EIF3D |
| GO:0090079 | translation regulator activity, nucleic acid binding | 4/57 | 108/18367 | 0.008 | DENR; EIF1B; EEF1A2; EIF3D |
| GO:0045182 | translation regulator activity | 4/57 | 140/18367 | 0.016 | DENR; EIF1B; EEF1A2; EIF3D |
| GO:0003785 | actin monomer binding | 3/57 | 28/18367 | 0.004 | COBL; LMOD2; MYL2 |
| GO:0003743 | translation initiation factor activity | 3/57 | 51/18367 | 0.010 | DENR; EIF1B; EIF3D |
| GO:0050660 | flavin adenine dinucleotide binding | 3/57 | 85/18367 | 0.037 | LDHD; CYB5R1; DLD |
| GO:0004738 | pyruvate dehydrogenase activity | 2/57 | 6/18367 | 0.004 | PDHX; DLD |
| GO:0034603 | pyruvate dehydrogenase [NAD(P)+] activity | 2/57 | 6/18367 | 0.004 | PDHX; DLD |
| GO:0034604 | pyruvate dehydrogenase (NAD+) activity | 2/57 | 6/18367 | 0.004 | PDHX; DLD |
| GO:0004364 | glutathione transferase activity | 2/57 | 26/18367 | 0.043 | GSTT2B; GSTM3 |
| *Molecular Functions - Incomplete Reverse Remodelling* | | | | | |
| GO:0004866 | endopeptidase inhibitor activity | 7/89 | 180/18367 | 0.004 | C4B; C4A; SERPINF2; ITIH4; C3; HRG; AGT |
| GO:0030414 | peptidase inhibitor activity | 7/89 | 187/18367 | 0.004 | C4B; C4A; SERPINF2; ITIH4; C3; HRG; AGT |
| GO:0061135 | endopeptidase regulator activity | 7/89 | 194/18367 | 0.004 | C4B; C4A; SERPINF2; ITIH4; C3; HRG; AGT |
| GO:0061134 | peptidase regulator activity | 7/89 | 230/18367 | 0.007 | C4B; C4A; SERPINF2; ITIH4; C3; HRG; AGT |
| GO:0045296 | cadherin binding | 7/89 | 332/18367 | 0.027 | CAPG; HDLBP; TNKS1BP1; TMOD3; RANBP1; KTN1; SEPTIN9 |
| GO:0003823 | antigen binding | 5/89 | 171/18367 | 0.029 | IGHV3-49; IGKV1-5; IGKV3-20; IGHG4; IGHM |
| GO:0005044 | scavenger receptor activity | 4/89 | 47/18367 | 0.006 | SSC5D; CD5L; LRP1; CD163 |
| GO:0038024 | cargo receptor activity | 4/89 | 77/18367 | 0.018 | SSC5D; CD5L; LRP1; CD163 |
| GO:0008135 | translation factor activity, RNA binding | 4/89 | 84/18367 | 0.020 | GSPT1; EIF3H; EEF1B2; EIF3B |
| GO:0004867 | serine-type endopeptidase inhibitor activity | 4/89 | 98/18367 | 0.028 | SERPINF2; ITIH4; HRG; AGT |
| GO:0090079 | translation regulator activity, nucleic acid binding | 4/89 | 108/18367 | 0.036 | GSPT1; EIF3H; EEF1B2; EIF3B |
| GO:0001848 | complement binding | 3/89 | 21/18367 | 0.007 | CRP; C4B; C4A |
| GO:0070325 | lipoprotein particle receptor binding | 3/89 | 28/18367 | 0.012 | CRP; LRP1; AP2A1 |
| GO:0016019 | peptidoglycan immune receptor activity | 2/89 | 5/18367 | 0.010 | PGLYRP2; CD14 |
| GO:0001849 | complement component C1q complex binding | 2/89 | 8/18367 | 0.019 | CRP; C4A |
| GO:0008641 | ubiquitin-like modifier activating enzyme activity | 2/89 | 10/18367 | 0.026 | NAE1; UBA1 |
| GO:0001846 | opsonin binding | 2/89 | 15/18367 | 0.042 | CRP; C4A |
| *Cellular Components – Complete Reverse Remodelling* | | | | | |
| GO:0005759 | mitochondrial matrix | 6/56 | 480/19549 | 0.034 | SUOX; TIMM44; PDHX; IARS2; DLD; OGDH |
| GO:1990204 | oxidoreductase complex | 5/56 | 112/19549 | 0.003 | GPD1; GMPR; PDHX; DLD; OGDH |
| GO:0030017 | sarcomere | 4/56 | 211/19549 | 0.039 | TNNI1; CAVIN4; LMOD2; MYL2 |
| GO:0031514 | motile cilium | 4/56 | 214/19549 | 0.039 | GSTM3; DDX6; SLIRP; DLD |
| GO:0030016 | myofibril | 4/56 | 231/19549 | 0.047 | TNNI1; CAVIN4; LMOD2; MYL2 |
| GO:0043292 | contractile fiber | 4/56 | 239/19549 | 0.047 | TNNI1; CAVIN4; LMOD2; MYL2 |
| GO:0001669 | acrosomal vesicle | 3/56 | 121/19549 | 0.047 | LOXL1; SLIRP; DLD |
| GO:0036126 | sperm flagellum | 3/56 | 122/19549 | 0.047 | GSTM3; DDX6; SLIRP |
| GO:0045252 | oxoglutarate dehydrogenase complex | 2/56 | 5/19549 | 0.007 | DLD; OGDH |
| GO:0031466 | Cul5-RING ubiquitin ligase complex | 2/56 | 7/19549 | 0.010 | CUL5; ELOB |
| GO:0045254 | pyruvate dehydrogenase complex | 2/56 | 8/19549 | 0.001 | PDHX; DLD |
| GO:0045240 | dihydrolipoyl dehydrogenase complex | 2/56 | 9/19549 | 0.010 | DLD; OGDH |
| GO:0045239 | tricarboxylic acid cycle enzyme complex | 2/56 | 13/19549 | 0.018 | DLD; OGDH |
| GO:0016282 | eukaryotic 43S preinitiation complex | 2/56 | 17/19549 | 0.026 | EIF1B; EIF3D |
| GO:0070993 | translation preinitiation complex | 2/56 | 18/19549 | 0.026 | EIF1B; EIF3D |
| GO:0005865 | striated muscle thin filament | 2/56 | 22/19549 | 0.030 | TNNI1; LMOD2 |
| GO:0022624 | proteasome accessory complex | 2/56 | 23/19549 | 0.032 | PSMD1; PSME2 |
| GO:0071782 | endoplasmic reticulum tubular network | 2/56 | 23/19549 | 0.032 | LNPK; RAB10 |
| GO:0036379 | myofilament | 2/56 | 26/19549 | 0.034 | TNNI1; LMOD2 |
| GO:0031672 | A band | 2/56 | 38/19549 | 0.047 | LMOD2; MYL2 |
| *Cellular Components – Incomplete Reverse Remodelling* | | | | | |
| GO:0072562 | blood microparticle | 19/90 | 146/19549 | <0.001 | C1QC; CD5L; IGKV1-5; IGKV3-20; C1QB; C1S; STOM; C4B; C4A; SERPINF2; IGHG4; ITIH4; IGHM; CFHR1; C4BPA; C3; HRG; CP; AGT |
| GO:0062023 | collagen-containing extracellular matrix | 10/90 | 425/19549 | 0.003 | SSC5D; C1QC; LTBP1; C1QB; MMP2; MMRN2; SERPINF2; ITIH4; HRG; AGT |
| GO:0034774 | secretory granule lumen | 7/90 | 322/19549 | 0.016 | SELENOP; SERPINF2; ITIH4; C3; HRG; DYNC1H1; PRDX4 |
| GO:0060205 | cytoplasmic vesicle lumen | 7/90 | 325/19549 | 0.016 | SELENOP; SERPINF2; ITIH4; C3; HRG; DYNC1H1; PRDX4 |
| GO:0031983 | vesicle lumen | 7/90 | 327/19549 | 0.016 | SELENOP; SERPINF2; ITIH4; C3; HRG; DYNC1H1; PRDX4 |
| GO:0019814 | immunoglobulin complex | 6/90 | 167/19549 | 0.005 | IGKV2-29; IGHV3-49; IGKV1-5; IGKV3-20; IGHG4; IGHM |
| GO:0042571 | immunoglobulin complex, circulating | 4/90 | 77/19549 | 0.013 | IGHV3-49; IGKV3-20; IGHG4; IGHM |
| GO:0042470 | melanosome | 4/90 | 109/19549 | 0.028 | RAB5C; CAPG; STOM; TMED10 |
| GO:0048770 | pigment granule | 4/90 | 109/19549 | 0.028 | RAB5C; CAPG; STOM; TMED10 |
| GO:0030140 | trans-Golgi network transport vesicle | 3/90 | 34/19549 | 0.014 | IGF2R; AP2A1; TMED10 |
| GO:0071753 | IgM immunoglobulin complex | 2/90 | 3/19549 | 0.003 | IGKV3-20; IGHM |
| GO:0071754 | IgM immunoglobulin complex, circulating | 2/90 | 3/19549 | 0.003 | IGKV3-20; IGHM |
| GO:0071756 | pentameric IgM immunoglobulin complex | 2/90 | 3/19549 | 0.003 | IGKV3-20; IGHM |
| GO:0071541 | eukaryotic translation initiation factor 3 complex, eIF3m | 2/90 | 7/19549 | 0.013 | EIF3H; EIF3B |
| GO:0031089 | platelet dense granule lumen | 2/90 | 14/19549 | 0.029 | SELENOP; ITIH4 |
| GO:0005852 | eukaryotic translation initiation factor 3 complex | 2/90 | 15/19549 | 0.030 | EIF3H; EIF3B |
| GO:0033290 | eukaryotic 48S preinitiation complex | 2/90 | 15/19549 | 0.030 | EIF3H; EIF3B |
| GO:0016282 | eukaryotic 43S preinitiation complex | 2/90 | 17/19549 | 0.036 | EIF3H; EIF3B |
| GO:0070993 | translation preinitiation complex | 2/90 | 18/19549 | 0.038 | EIF3H; EIF3B |
| GO:0042827 | platelet dense granule | 2/90 | 21/19549 | 0.050 | SELENOP; ITIH4 |

**Table S3.** Complete Kinase Rank, containing all predicted kinases and kinase families and the respective percentage of phosphorylation events in complete and incomplete reverse remodelling.

| **Predicted Kinase/Family** | **Family** | **Kinase/Kinase Group** | **% Phosphorylation events** | | |
| --- | --- | --- | --- | --- | --- |
|  |  |  | **cRR** | **iRR** | **∆** |
| CMGC/DYRK/DYRK1/DYRK1A | CMGC | Dual specificity tyrosine-phosphorylation-regulated kinase 1A | 2.5% | 0.7% | -1.9% |
| CMGC/DYRK/DYRK2 | CMGC | Dual specificity tyrosine-phosphorylation-regulated kinase 2 | 2.1% | 0.4% | -1.7% |
| TKL | TKL | - *Vide infra* | 3.2% | 1.8% | -1.4% |
| CMGC/MAPK | CMGC | Mitogen-Activated Protein Kinase | 2.5% | 1.3% | -1.2% |
| TKL/MLK | TKL | Mixed Lineage Kinases | 3.0% | 2.1% | -0.9% |
| CMGC/GSK | CMGC | Glycogen Synthase Kinase | 4.8% | 4.0% | -0.9% |
| CAMK/CAMK-Unique | CAMK | Unique | 0.9% | 0.1% | -0.8% |
| CAMK/PIM | CAMK | Pim | 0.7% | 0.0% | -0.7% |
| Other/CAMKK | Other | CAM kinase kinase | 0.7% | 0.0% | -0.7% |
| Other/WEE | Other | Wee | 0.7% | 0.0% | -0.7% |
| TK/VEGFR | TK | Vascular Endothelial Growth Factor Receptor | 0.7% | 0.1% | -0.6% |
| TKL/RIPK | TKL | Receptor Interacting Protein Kinases | 2.8% | 2.2% | -0.5% |
| AGC/SGK | AGC | Serum and Glucocorticoid induced Kinases | 0.5% | 0.0% | -0.5% |
| CAMK/CAMKL | CAMK | CAM kinase-like | 0.7% | 0.3% | -0.4% |
| STE | STE | - *Vide infra* | 0.7% | 0.3% | -0.4% |
| CAMK/CAMK1 | CAMK | Calcium/calmodulin-dependent protein kinase type 1 | 1.1% | 0.8% | -0.4% |
| STE/STE7/MEK1/MAP2K2 | STE | Dual specificity mitogen-activated protein kinase kinase 2 | 0.5% | 0.1% | -0.3% |
| TK/Syk | TK | Spleen tyrosine kinase | 0.5% | 0.1% | -0.3% |
| CK1/TTBK | CK1 | Tau-Tubulin Kinase | 0.9% | 0.7% | -0.3% |
| CAMK/MLCK | CAMK | Myosin Light Chain Kinase | 0.2% | 0.0% | -0.2% |
| TK/Alk | TK | Anaplastic Lymphoma Kinase | 0.2% | 0.0% | -0.2% |
| TK/Csk | TK | C-terminal Src kinases | 0.2% | 0.0% | -0.2% |
| TK/PDGFR | TK | Platelet-Derived Growth Factor Receptor | 0.2% | 0.0% | -0.2% |
| Other/MOS | Other | Mos | 0.5% | 0.3% | -0.2% |
| TK | TK | - *Vide infra* | 0.5% | 0.3% | -0.2% |
| AGC/PKG | AGC | Protein Kinase G | 0.7% | 0.5% | -0.2% |
| CAMK/CASK | CAMK | Calcium/calmodulin-dependent serine protein kinase | 0.7% | 0.5% | -0.2% |
| CAMK/PKD | CAMK | Protein Kinase D | 0.7% | 0.5% | -0.2% |
| AGC/GRK | AGC | G-protein-coupled receptor kinases | 2.5% | 2.4% | -0.1% |
| Atypical/Alpha | Atypical | Alpha | 0.2% | 0.1% | -0.1% |
| CAMK/RAD53 | CAMK | Checkpoint kinase 2 | 0.2% | 0.1% | -0.1% |
| Other/TLK | Other | Tousled-Like Kinase | 0.2% | 0.1% | -0.1% |
| Other/WNK | Other | "With no K (Lysine)" kinase | 0.2% | 0.1% | -0.1% |
| STE/STE7/MEK3/MAP2K6 | STE | Dual specificity mitogen-activated protein kinase kinase 6 | 0.2% | 0.1% | -0.1% |
| TK/Jak | TK | Janus Kinases | 0.2% | 0.1% | -0.1% |
| TK/Src | TK | Src family | 0.2% | 0.1% | -0.1% |
| TKL/STKR/STKR2/TGFbR2 | TKL | Transforming growth factor-beta receptor type 2 | 1.1% | 1.1% | -0.1% |
| AGC/NDR | AGC | NDR family | 0.5% | 0.4% | -0.1% |
| CAMK/CAMK2 | CAMK | Calcium/calmodulin-dependent protein kinase type 2 | 0.5% | 0.4% | -0.1% |
| CK1 | CK1 | - *Vide infra* | 0.5% | 0.4% | -0.1% |
| CMGC/CDK | CMGC | Cyclin Dependent Kinases | 1.4% | 1.3% | -0.1% |
| CMGC/DYRK | CMGC | Dual specificity Tyrosine Regulated Kinase | 0.7% | 0.7% | 0.0% |
| Other/ULK | Other | Unc-51 like kinase | 0.7% | 0.7% | 0.0% |
| AGC/PDK1 | AGC | 3-phosphoinositide-dependent protein kinase 1 | 5.3% | 5.3% | 0.0% |
| Other/PEK | Other | PEK family | 2.8% | 2.8% | 0.0% |
| Other/Bud32 | Other | Bud32 family | 0.2% | 0.3% | 0.0% |
| AGC/PKA | AGC | Protein Kinase A | 0.5% | 0.5% | 0.1% |
| STE/STE7 | STE | STE7 | 0.5% | 0.5% | 0.1% |
| STE/STE7/MEK1/MAP2K1 | STE | Dual specificity mitogen-activated protein kinase kinase 1 | 2.3% | 2.4% | 0.1% |
| Atypical/PDHK | Atypical | Pyruvate Dehydrogenase Kinases | 0.0% | 0.1% | 0.1% |
| Other/Aur | Other | Aurora family | 0.0% | 0.1% | 0.1% |
| STE/STE7/MEK3/MAP2K3 | STE | Dual specificity mitogen-activated protein kinase kinase 3 | 0.0% | 0.1% | 0.1% |
| TK/Eph | TK | Ephrin receptors | 0.0% | 0.1% | 0.1% |
| TKL/IRAK | TKL | Interleukin 1 Receptor Associated Kinase | 0.0% | 0.1% | 0.1% |
| TKL/STKR/STKR2 | TKL | Serine Threonine Kinase Receptors Type 2 | 0.0% | 0.1% | 0.1% |
| STE/STE11 | STE | STE11 | 0.2% | 0.4% | 0.2% |
| STE/STE20 | STE | STE20 | 0.2% | 0.4% | 0.2% |
| STE/STE7/MEK3/MAP2K4 | STE | Dual specificity mitogen-activated protein kinase kinase 4 | 0.2% | 0.4% | 0.2% |
| Other/TTK | Other | Tau Tubulin Kinase | 0.0% | 0.3% | 0.3% |
| AGC/DMPK | AGC | Myotonic Dystrophy Protein Kinase | 1.8% | 2.1% | 0.3% |
| AGC/PKN | AGC | Protein Kinase N | 0.2% | 0.5% | 0.3% |
| Atypical/PIKK | Atypical | Phosphatidyl inositol 3’ kinase-related kinases | 0.2% | 0.5% | 0.3% |
| TKL/LISK | TKL | LISK family | 0.2% | 0.5% | 0.3% |
| AGC/RSK | AGC | Ribosomal protein S6 Kinases | 1.6% | 2.0% | 0.4% |
| Other/PLK | Other | Polo-Like Kinases | 5.3% | 5.7% | 0.4% |
| STE/STE-Unique | STE | Unique | 0.2% | 0.7% | 0.4% |
| Other/CDC7 | Other | Cell division cycle 7-related protein kinase | 0.5% | 0.9% | 0.5% |
| AGC/PKC | AGC | Protein Kinase C | 9.6% | 10.2% | 0.5% |
| CMGC/DYRK/DYRK1 | CMGC | Dual specificity tyrosine-phosphorylation-regulated kinase 1A | 0.0% | 0.5% | 0.5% |
| CK1/CK1 | CK1 | Casein Kinase 1 Family | 9.2% | 9.8% | 0.6% |
| CMGC/CLK | CMGC | CDC-like kinase 1 family | 0.7% | 1.3% | 0.6% |
| TKL/RAF | TKL | Raf family | 0.9% | 1.6% | 0.7% |
| Other/NEK | Other | Nek family | 0.2% | 1.1% | 0.8% |
| CAMK/MAPKAPK | CAMK | MAPK-Activated Protein Kinase | 3.0% | 3.8% | 0.8% |
| CAMK/PHK | CAMK | Phosphorylase Kinase | 5.0% | 5.9% | 0.9% |
| CK1/VRK | CK1 | Vaccinia Related Kinase | 2.8% | 3.7% | 0.9% |
| Other/IKK | Other | I-kappa kinase | 1.1% | 2.1% | 1.0% |
| TKL/STKR | TKL | Serine Threonine Kinase Receptors | 0.5% | 1.8% | 1.4% |
| Atypical/TAF1 | Atypical | Transcription initiation factor TFIID subunit 1 | 0.7% | 2.5% | 1.8% |
| CMGC/CK2 | CMGC | Casein Kinase 2 | 1.4% | 3.4% | 2.1% |
| **Kinase Families** | | | | | |
| AGC | Named after the Protein Kinase A, G, and C families (PKA, PKG, PKC) | | | | |
| Atypical | Set of diverse kinases with no structural similarity with the remaining eukaryotic protein kinases | | | | |
| CAMK | Calcium/calmodulin regulated kinases | | | | |
| CMGC | Named after the CDK, MAPK, GSK3 and CLK families | | | | |
| CK1 | Casein Kinase 1/Cell Kinase 1 family | | | | |
| Other | Set of unique kinases that do not belong to the remaining categories | | | | |
| PKL | Protein Kinase-Like family | | | | |
| RGC | Receptor Guanylate Cyclases | | | | |
| STE | Homologs of the yeast STE7, STE11, and STE20 genes, which form the MAPK cascade | | | | |
| TK | Tyrosine Kinase family, which targets Tyr residues | | | | |
| TKL | Tyrosine kinase-like family, similar to TK, but targeting Ser/Thr | | | | |
